# Itacitinib in advanced hepatocellular cancer following first line therapy

**DOI:** 10.1038/s41698-026-01273-9

**Published:** 2026-02-11

**Authors:** Alessandro Troiani, Lingshan Hung, David J. Pinato, Maria Martinez, Debashish Sarker, Caroline Ward, Hooshang Izadi, Rob Goldin, Mathew Vithayathil, Sultan Alharbi, Haonan Lu, Rohini Sharma

**Affiliations:** 1 Division of Surgery and Cancer, Imperial College London, Hammersmith Hospital Campus, London, UK; 2 Department of Translational Medicine, Università Del Piemonte Orientale, Novara, Italy; 3 Department of Medical Oncology, King’s College London, London, UK; 4 Department of Mechanical Engineering and Mathematical Sciences, Oxford Brookes University, Oxford, UK; 5 Department of Metabolism, Digestion and Reproduction, Imperial College London, Hammersmith Hospital, London, UK; 6 Department of Obstetrics and Gynaecology, LKS Faculty of Medicine, The University of Hong Kong, Pok Fu Lam, Hong Kong; 7 Materials Innovation Institute for Life Sciences and Energy (MILES), HKU-SIRI, Shenzhen, PR China; 8 Shenzhen Key Laboratory of Fertility Regulation, Center of Assisted Reproduction and Embryology, The University of Hong Kong-Shenzhen Hospital, Shenzhen, PR China

**Keywords:** Medical research, Drug development, Hepatocellular carcinoma

## Abstract

Hepatocellular carcinoma (HCC) develops on background chronic liver disease where uncontrolled inflammation drives hepatocarcinogenesis. First-line therapies have limited response. There are no agents that specifically target the underlying liver inflammation. We investigated the safety and efficacy of itacitinib, a highly selective JAK1 inhibitor, as a potential second- or third-line therapy. Biomarkers of response were investigated. Participants with advanced stage HCC, Child Pugh ≤B7 who had progressed through at least one previous line of therapy received 400 mg of itacitinib QID every 28 days. Treatment-related adverse events (trAEs) were assessed weekly during cycle 1 then every 28 days (CTC-AE version 4.03). Response was assessed 8-weekly using RECIST 1.1. Progression-free (PFS) and overall survival (OS) were reported as secondary endpoints. Tumour samples were analysed for targeted transcriptomics. Sequential serum samples were assessed for metabonomic determinants of toxicity and response. 19 patients were enrolled. The most common trAEs were thrombocytopenia (31%), fatigue (26%) and palmar-plantar erythrodysesthesia syndrome (26%); four episodes of dose-limiting thrombocytopaenia were observed. Over a median follow-up of 3.5 months, the best overall response was stable disease (47%). Median PFS and OS were 3.5 (95% CI: 2.6 - 4.5 months) and 7.4 months (95% CI: 4.3-10.5 months), respectively. Subgroup analysis illustrated a significantly increased risk of progression for patients that had received combination immunotherapy prior to itacitinib (HR 4.7, 95%CI 1.3-16.6, *p* = 0.016). Untargeted tumour and serum transcriptomics identified a signature predictive of response. Itacitinib either as second- or third-line therapy showed promising activity. We identified a transcriptomic signature predictive of response.

## Introduction

Hepatocellular carcinoma (HCC) develops on a background of chronic liver inflammation secondary to infection with hepatotropic viruses, alcohol excess and increasingly metabolic dysfunction-associated steatotic liver disease^1^. The mechanisms through which the above conditions trigger the development of cirrhosis and HCC involve persistent inflammation and immune cell infiltration of the hepatic parenchyma^2,3^. The sustained exposure to IL-6, TNF-α, reactive oxygen species and other cytokines released by monocytes and T-cells leads to cell death and regeneration of hepatocytes^4^. The repeated damage and repair of the hepatic tissues give rise to fibrotic changes and lipid accumulation within the parenchyma^5^. Intracellularly, the same processes may lead to the accumulation of DNA mutations or epigenetic changes, such as upregulation of telomerase complexes to prevent hepatocyte senescence^6,7^. It is these alterations of the hepatic genome, microenvironment and architecture that precipitate the malignant transformation of local cells and the development of HCC^7^.

Janus Kinase and Signal Transducer and Activator of Transcription (JAK/STAT) signalling is central to the pathogenesis of HCC, which is driven by persistent hepatocyte exposure to the pro-inflammatory cytokine IL-6^8,9^. On binding of IL-6 to its receptor, the JAK protein family (JAK1, JAK2, JAK3 and TYK2) are activated, resulting in subsequent phosphorylation and activation of the downstream STAT proteins^8^. Overactivation of the JAK/STAT pathway promotes malignant transformation of inflamed hepatocytes, as well as contributing to tumour growth and progression, and as such is a promising therapeutic target^8^.

Combination immunotherapy remains the mainstay of treatment for advanced HCC^10^. However, the majority of patients will eventually progress, and whilst the tyrosine kinase inhibitors, lenvatinib and sorafenib, are recommended second-line therapies, there is a paucity of evidence for their therapeutic efficacy in this setting^11^. Moreover, these therapies are only available to patients with maintained liver function (Child-Pugh class A), and the utility of systemic therapy in patients with Child-Pugh B disease has not been proven prospectively^12,13^. Most patients with HCC have cirrhosis, such that patients have two competing co-morbidities, and whilst systemic therapy will address progression of the malignancy, no agent has been shown to slow or delay the progression of the underlying chronic inflammation.

Due to the role of the JAK/STAT pathway in HCC development and progression, it is hypothesised that inhibition of the pathway could lead to prolonged survival in patients with advanced-stage HCC and delay the progression of chronic liver disease. JAK inhibition may have direct anti-tumour activity in HCC, as 10% of HCC harbour a mutation in JAK1^14^. Moreover, activation of the JAK/STAT pathway plays a key role in creating an immunosuppressive tumour microenvironment, and blockade may therefore improve clinical outcome^15^. We report the results of a phase 1b trial investigating the safety and efficacy of itacitinib as second/third-line systemic therapy in patients with advanced HCC. Itacitinib is a specific JAK1 inhibitor that relatively spares JAK2 signalling and may have less haematologic side-effects, in particular thrombocytopaenia, a key consideration in patients with CLD^16^. Previous studies of JAK inhibitors have stratified treatment cohorts according to CRP, which failed as a pharmacodynamic marker^17^. We therefore undertook an untargeted transcriptomics approach on both tissue and blood to identify potential biomarkers of clinical outcome.

## Results

### Participant cohort

**Fig. 1:**
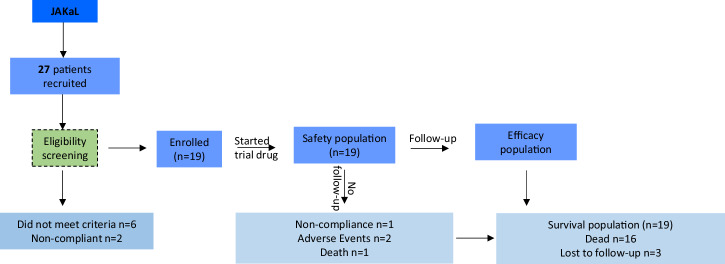
Flow diagram of the JAKaL trial participant cohort. Recruitment began in January 2019 and was stopped in December 2022. Data cut-off was 30/04/2023.

Between January 2019 and December 2022, 27 patients were consented and screened for study entry. Of these, 19 participants were deemed eligible and received at least one dose of itacitinib and were included in the safety analysis. In terms of efficacy analysis, four participants did not undergo radiologic evaluation (one patient was withdrawn from the study due to lack of compliance, two experienced severe AEs that led to treatment cessation, and one died prior to response assessment). Therefore, 15 patients were included in the efficacy analysis (Fig. 1).

The median age of the population was 66 years (range 62–73). Most patients had Child-Pugh A cirrhosis (79%) secondary to HCV (37%). Four (21%) patients had CP-B7 liver dysfunction at enrolment. The patient cohort was heavily pretreated, with 79% of patients having received at least two previous therapies (range 1–5). Eleven (58%) patients received itacitinib as second-line therapy, five of whom had received combination immunotherapy with atezolizumab and bevacizumab as first-line systemic therapy. Patient demographics are summarised in Table 1.

**Table 1:** Participant demographics (*n* = 19)

	Enroled patients
**Age in years**, median (range)	66 (62–73)
**Baseline serum AFP in µg/L**, median (range)	7 (4-226)
**Sex,** ***n*** **(%)**	
*M*	18 (94.7)
**Cirrhosis,** ***n*** **(%)**	
Present	18 (94.7)
**Cause of cirrhosis,** ***n*** **(%)**	
HBV	3 (15.8)
HCV	7 (36.8)
ALD	4 (21.1)
MASLD	5 (26.3)
**Child-Pugh class,** ***n*** **(%)**	
A	15 (78.9)
B	4 (21.1)
**BCLC stage**	
B	7 (36.8)
C	12 (63.2)
**Extrahepatic metastasis,** ***n*** **(%)**	
Lung	2 (10.5)
Bone	3 (15.8)
Lymph nodes	10 (52.6)
Peritoneal	4 (21.1)
None	6 (31.6)
Other	1 (5.3)
**PVT,** ***n*** **(%)**	
Present	7 (36.8)
Not present	12 (63.2)
**Baseline ECOG PS,** ***n*** **(%)**	
0	11 (57.9)
1	8 (42.1)
**Previous treatments,** ***n*** **(%)**	
Surgery	5 (26.3)
TACE	10 (52.6)
MWA/RFA	7 (31.6)
SIRT	2 (10.5)
Sorafenib	12 (63.2)
Lenvatinib	4 (21.1)
Regorafenib	5 (26.3)
Atezolizumab/Bevacizumab	5 (26.3)
Other	5 (26.3)

*AFP* alpha-fetoprotein, *BMI* Body mass index, *HBV/HCV* Hepatitis B/C virus, *ALD* alcohol related liver disease, *MASLD* metabolic dysfunction-associated steatotic liver disease, *BCLC* Barcelona Clinic Liver Cancer, *PVT* portal vein thrombosis, *ECOG PS* Eastern Cooperative Oncology Group Performance Score, *TACE* transarterial chemoembolisation, *MWA* microwave ablation, *RFA* radiofrequency ablation, *SIRT* selective internal radiation therapy.

### Safety analysis

TrAEs were experienced by 74% of participants, the most common trAE of any grade were thrombocytopenia (31%), fatigue (26%) and palmar-plantar erythrodysesthesia syndrome (26%). Table 2 summarises all trAEs. In terms of dose-limiting toxicity (DLT), four episodes of thrombocytopaenia occurred in two patients, resulting in treatment discontinuation. In terms of dose reductions, four patients (21%) had a dose reduction; two subjects developed grade 3 thrombocytopaenia, 1 patient developed raised transaminases, and one patient acutely decompensated. No episodes of febrile neutropenia were observed. No toxicity-related deaths were noted. When considering patients with Child-Pugh B liver dysfunction (*n* = 4), one patient discontinued itacitinib due to grade 4 thrombocytopaenia, and two patients experienced treatment interruption for raised LFTs and myelosuppression. No increase in adverse event rate was observed in this patient cohort (median 3, range 1–6) compared to those with Child-Pugh A liver dysfunction (median 2, range 0–7).

**Table 2:** Summary of all treatment-related adverse events (*n* = 19) according to NCI CTC-AE version 4.03

	Number of TRAEs (% of patients affected)	
	Any grade	Grade ≥ 3
Thrombocytopaenia	7 (31.6)	4 (21.1)
Fatigue	5 (26.3)	0 (0.0)
PPE	5 (26.3)	0 (0.0)
Anaemia	4 (21.1)	1 (5.3)
Diarrhoea	3 (10.5)	0 (0.0)
Mucositis	3 (15.8)	0 (0.0)
Myelosuppression	2 (10.5)	0 (0.0)
Hypertension	2 (10.5)	0 (0.0)
Nausea/Vomiting	2 (10.5)	0 (0.0)
Neutropaenia	2 (10.5)	0 (0.0)
Raised LFTs	2 (10.5)	2 (10.5)
Anorexia	1 (5.3)	0 (0.0)
Dysgeusia	1 (5.3)	0 (0.0)
Insomnia	1 (5.3)	0 (0.0)
**Total patients with AEs (%)**	14 (73.7)	6 (31.6)

*TRAEs* treatment-related adverse events, *PPES* palmar-plantar erythrodysesthesia syndrome, *LFTs* liver function tests, *AE* adverse event.

### Efficacy

**Fig. 2:**
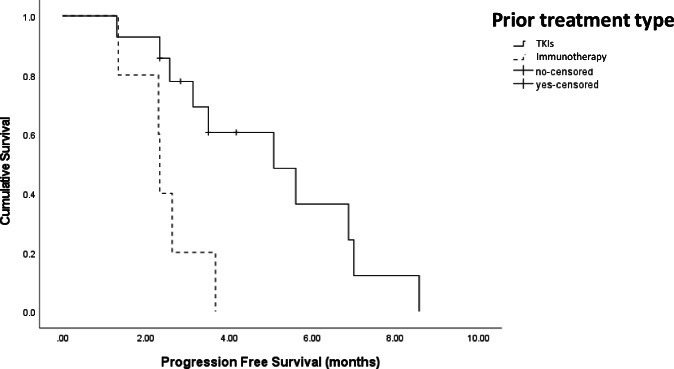
Kaplan–Meier curve with log-rank analysis of participants’ PFS stratified based on first-line systemic therapy, either tyrosine kinase inhibitors or atezolizumab and bevacizumab combination.

The median exposure to itacitinib was 2.6 months (range 0.73–8.4 months), and the mean dose intensity administered was 2260 mg/week. The best overall response was SD observed in 47% (*n* = 7), whilst the rest experienced PD (*n* = 8, 53%). No subject experienced a partial or complete response. The median PFS was 3.5 months (95% CI: 2.6–4.5 months). Patients who experienced disease had a significantly longer PFS (6.9 months, 95% CI: 5.5–8.2) compared to those who experienced PD (2.5 months, 95% CI: 2.1–2.6, *p* < 0.001). Patients that had received combination immunotherapy first-line therapy with atezolizumab/bevacizumab had a worse median PFS (2.3 months) compared to those who received a TKI first line (median PFS 5.1 months, HR 4.7, 95% CI: 1.3–16.6, *p* = 0.02) (Fig. 2). No other variables including number of previous lines of systemic therapy, presence of extrahepatic metastases, or baseline AFP had an impact on PFS on univariate analysis.

Three patients received subsequent therapy after itacitinib: regorafenib (*n* = 2) and lenvatinib (*n* = 1). The median OS for the entire patient cohort was 7.4 months (95% CI: 4.3–10.5 months). No difference was observed in OS in those with Child-Pugh B liver dysfunction compared to A, nor in those who received itacitinib second or third line (6.4 months, 95% CI: 3.0–9.4 versus 8.9 months, 95% CI: 1.5–16.2, *p* = 0.8).

### Targeted transcriptomics of baseline tumour biopsies

**Fig. 3:**
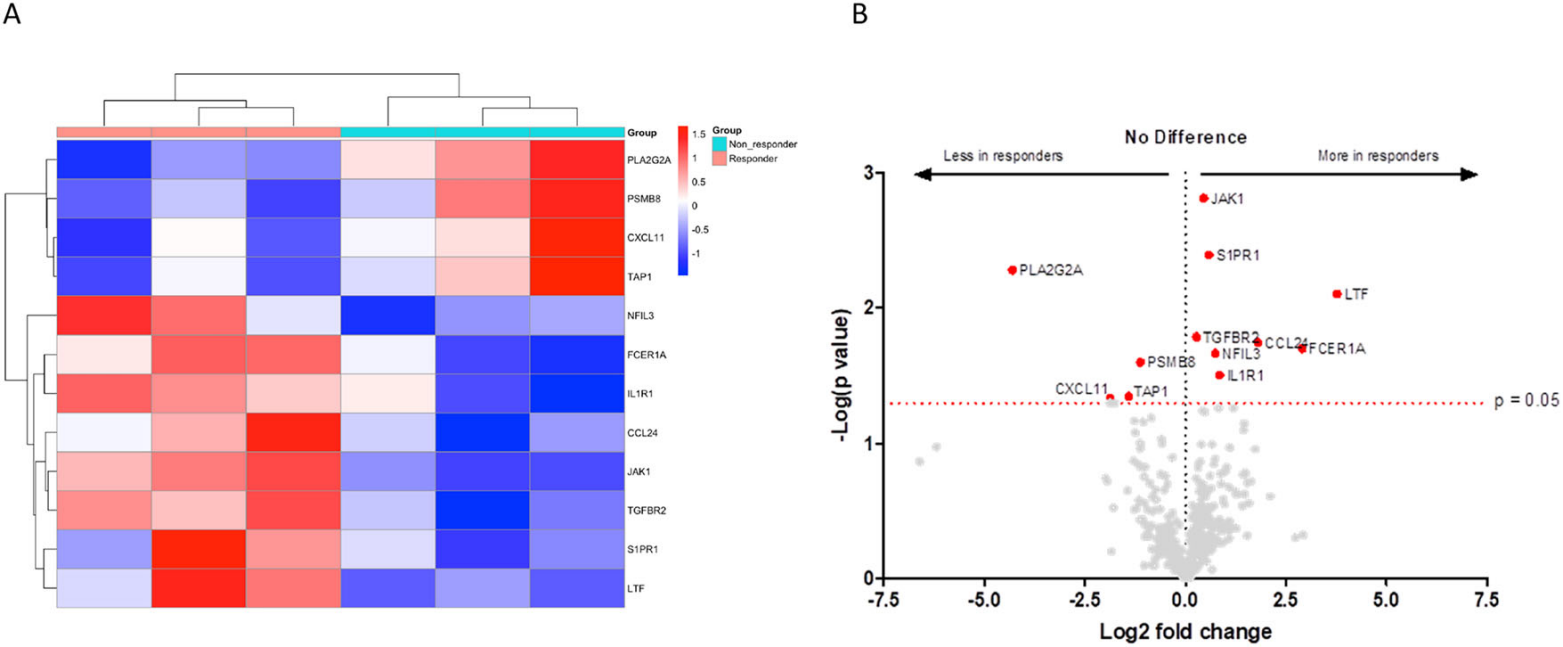
Tumour transcriptomics illustrate differential gene expression between responders and non-responders. **A** Heatmap showing the most significantly differentially expressed genes in responders (*n* = 3) compared to non-responders (*n* = 3). Rows correspond to genes, and columns represent individual samples. The heatmap uses a red-to-blue gradient to reflect relative gene expression levels, with red denoting upregulation and blue downregulation. Gene selection was based on an adjusted p-value threshold of <0.05 and a fold change of ≥1.5. Hierarchical clustering highlights similar expression patterns, distinguishing responders from non-responders. **B** The volcano plot illustrates the distribution of differentially expressed genes between responders (*n* = 3) and non-responders (*n* = 3), as analysed using the Nanostring Human Immunology panel. The *x*-axis represents the log₂ fold change, and the *y*-axis denotes the −log₁₀ (*p* value). Genes with significant differential expression (adjusted *p* value < 0.05) are highlighted in red. Genes located on the left side of the plot are expressed at lower levels in responders (i.e. more abundantly expressed in non-responders). In contrast, those on the right are upregulated in responders. The horizontal dashed line indicates the significance threshold (*p* = 0.05). This plot visually represents the magnitude and significance of gene expression changes between the two groups.

Bulk targeted transcriptomic analyses were performed on total RNA purified from six pretreatment biopsies obtained at screening that satisfied quality control criteria. None of these patients had received combination immunotherapy as first-line. In an exploratory analysis, we compared differences in the expression of 579 genes related to adaptive and innate immunity in 3 responders and 3 non-responders and illustrates a clear difference between the two groups (Supplementary Fig. 1). Gene set enrichment analysis (GSEA) illustrated that samples from patients achieving a response were enriched for gene expression signatures reflective of innate immunity including cytokine and chemokine secretion whilst downregulation was observed in gene signatures reflective of antigen presentation (Supplementary Fig. 2). In particular, differential gene expression analysis highlighted transcripts involved in the inflammatory process in particular JAK1, CCL24, TGFR-β and IL-1R1 were upregulated in responders compared to non-responders (Fig. 3A, B). Whilst a downregulation in phospholipase A2 and expression of genes responsible for antigen presentation (PSMB8 and TAP1) and chemotaxis of CD8+ T cells into the tumour microenvironment (CXCL11) was observed (Fig. 3A, B).

### Metabolic phenotyping predicts response to itacitinib

**Fig. 4:**
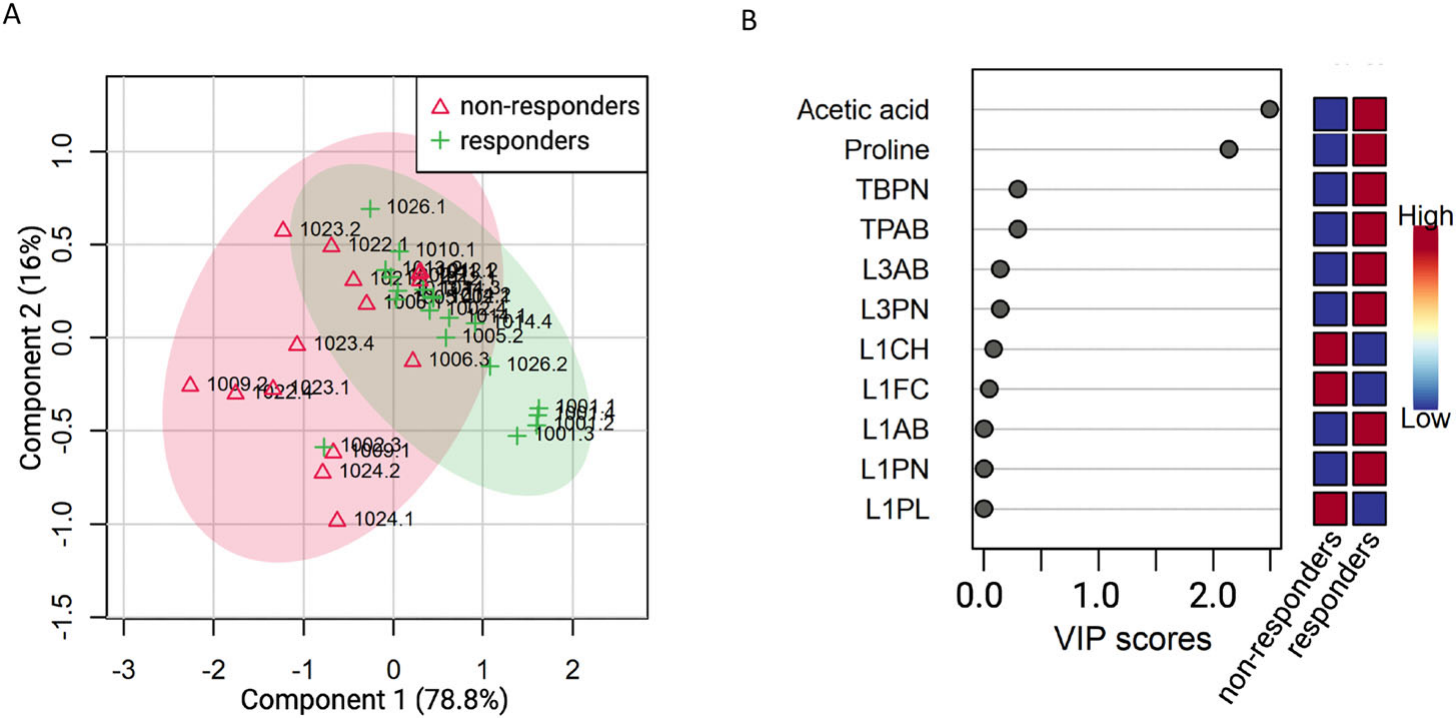
Metabolic phenotyping illustrates metabolic profile predictive of response. **A** Partial least squares discriminant analysis (PLS-DA) plot showing the separation of samples. The first component explains 78.8% of the variance, and the second component explains 16%. Red triangles for non-response and green crosses, response. The ellipses represent 95% confidence intervals for each group. **B** Variable Importance in Projection (VIP) scores for the top metabolites. Metabolites are ordered by their VIP scores, with higher scores indicating greater importance in distinguishing between non-responders and responders. *Indicates metabolites with significant VIP scores (VIP > 1.5).

**Fig. 5:**
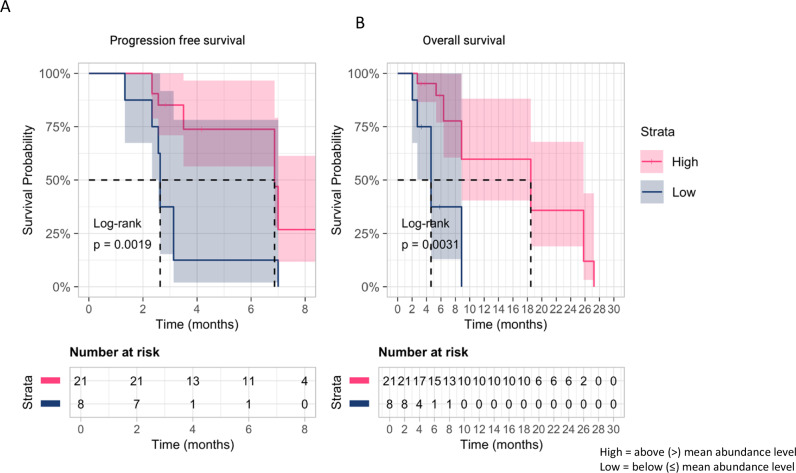
Metabolic phenotyping illustrates individual metabolites of survival outcomes. Kaplan–Meier survival curves for **A** proline and **B** acetic acid levels, separated into high and low groups based on the median value. The plots show the survival probability over time (in months), with log-rank *p* values indicating the significance of the differences between groups. The number of subjects at risk is displayed below each plot for different time points. Strata are colour-coded: high (red), low (blue).

Following normalisation, we undertook a PLS regression approach and observed that relative changes in metabolites differentiated response to itacitinib (Supplementary Figs. 3 and 4). Differential expression of 11 metabolites predicted for treatment response; an increase in acetic acid and proline and a reduction in the relative abundance of L1AB, L1CH, L1FC, L1PL, L1PN, L3AB, L3PN, TBPN, and TPAB was observed as being predictive of response to itacitinib (Fig. 4B). On visual inspection, there was a reduction in abundance of both proline and acetic acid in those experiencing a treatment response which increased at disease progression, compared to those with no response where no change was observed (Supplementary Figs. 5–8). We then investigated whether baseline proline or acetic acid levels were predictive of clinical outcome. Median proline was predictive of PFS with a high level predicting for poor survival compared with low levels (HR = 3.941, 95% CI = 1.546–10.05, *p* = 0.002; Fig. 5A). Similarly, high serum proline predicted for OS (HR = 5.926, 95% CI = 1.711–20.52, *p* = 0.003; Fig. 5B). Acetic acid was not predictive of survival outcomes.

**Fig. 6:**
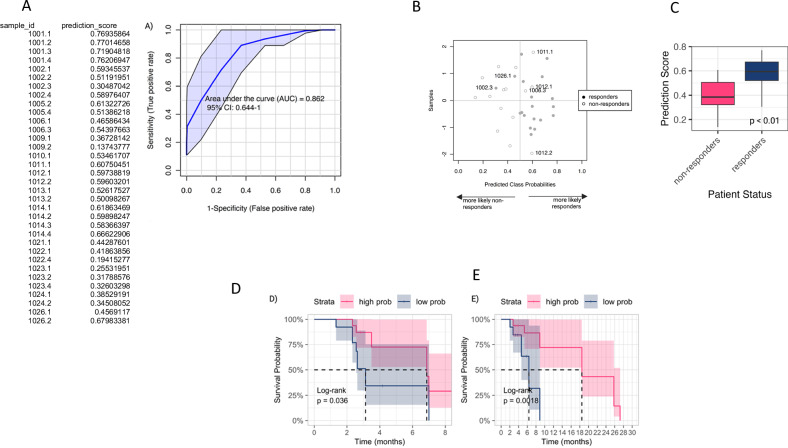
Metabolic phenotyping illustrates composite metabolic profile predictive of clinical outcomes. Multivariate Exploratory ROC Analysis on PLS-DA model: **A** ROC curve plot for the best performing model based on its average performance across all Monte-Carlo cross-validation runs. **B** Plot of the predicted class probabilities of all samples using the model described in (**A**), where wrongly classified samples are labelled. The probability scores for each sample are displayed in the left table. **C** Prediction score accurately classified responders and non-responders. Wilcoxon rank-sum test. Kaplan–Meier survival curves using prediction score to assess **D** PFS and **E** OS. The plots show the survival probability over time (in months), with log-rank *p* values indicating the significance of the differences between groups. The number of subjects at risk is displayed below each plot for different time points. Strata are colour-coded: high (red), low (blue).

Using a PLS regression approach, we combined the relative changes in 11 metabolites to create a prediction score that was derived for each individual in the study (Fig. 6A, B). The prediction score was significantly elevated in those with response compared to non-responders (*p* < 0.01, Fig. 6C) and was predictive of both PFS (HR = 0.9360, 95% CI = 1.03–6.313, *p* = 0.04; Fig. 6D) and OS (HR = 1.9969, 95% CI = 1.983–27.36, *p* = 0.002; Fig. 6E).

## Discussion

Considerable advances have been achieved in recent years in the treatment of advanced HCC, with the approval of combination immunotherapy in the first-line setting^18^. However, most patients will eventually relapse, and there is a lack of randomised controlled trial evidence guiding the choice of second-line therapy. There is also a paucity of prospective studies investigating the safety and efficacy of therapies in patients with impaired liver function.

Overactivation of the JAK/STAT pathway is a common feature in the pathophysiology of solid malignancies, including HCC^9^ where persistent activation of STAT3 results in the over-expression of anti-apoptotic and pro-angiogenic pathways^19^. This has led to considerable interest in JAK inhibitors as potential therapies for solid tumours. However, trials thus far have been disappointing, with no one inhibitor entering routine clinical management of solid tumours. We assessed the utility of the selective JAK1 inhibitor, itacitinib, as a second/third-line option for patients with advanced HCC with CP-A/B liver dysfunction. We have shown a response to itacitinib results in PFS similar to regorafenib and cabozantinib^20,21^. Moreover, we have illustrated that both tissue and circulating biomarkers can predict outcome to therapy, a key aspect omitted from previous trials of JAK inhibitors in solid tumours.

The safety profile of itacitinib is consistent with previous studies, the most common non-haematological trAEs being palmar-plantar erythrodysesthesia syndrome, fatigue and diarrhoea^22–24^. While rates of anaemia were also comparable to those observed in previous literature, we report a significantly higher rate of grade 4/5 thrombocytopaenia; one patient was subsequently diagnosed with immune thrombocytopaenia, possibly induced by itacitinib. In previous studies with ruxolitinib, an inhibitor of both JAK1 and JAK2, anaemia and thrombocytopaenia were observed, consistent with the role of JAK2 in erythropoiesis and thrombopoiesis^25,26^. However, as itacitinib is a specific JAK1 inhibitor, the rates of severe thrombocytopaenia are unexpected. Three out of four participants with Child-Pugh B liver dysfunction experienced severe trAEs. Itacitinib is predominantly metabolised in the liver by CYP 3A4 P450, and it has been demonstrated that exposure to itacitinib is doubled in patients with moderate liver dysfunction and 4-fold in those with severe dysfunction, suggesting that dose reduction or exclusion of patients with liver dysfunction in future studies^27^.

Survival analysis illustrated a median PFS of 5.3 months, comparable to data supporting currently available second-line therapies following sorafenib. Both RESORCE (regorafenib) and CELESTIAL (cabozantinib) phase 3 trials report a median PFS of 3.1 and 5.2 months, respectively^20,21^. However, itacitinib showed only modest OS benefit, consistent with the placebo arm of both the RESORCE and CELESTIAL studies. This discrepancy may relate to the trial population, whereby patients in our study had been heavily pretreated prior to enrolment with the inclusion of patients with liver dysfunction. However, other JAK inhibitors, such as ruxolitinib, have encountered similarly discouraging outcomes in the management of solid tumours^26^. Previous work has suggested that the activation of compensatory pathways, such as the PI3K/Akt/mTOR pathway, bypass JAK, may account for the lack of significant survival benefit observed^8,28^. Preclinical studies have observed that isolated JAK inhibition suppressed growth but was not able to induce apoptosis, suggesting that itacitinib monotherapy is insufficient. There has been recent interest in combining JAK inhibitors with immunotherapy to counter the negative role of persistent inflammation in the tumour microenvironment, an issue particularly relevant in HCC^29^.

The objective response to combination immunotherapy with atezolizumab and bevacizumab is only 27%^18^. Chronic inflammation, driven by IFNs, results in CD8+ T cell exhaustion and poor response to immunotherapy. Moreover, tumours harbouring *JAK1* deletion will avoid immunosurveillance, a key consideration in HCC where up to 10% of cases have a *JAK1* mutation^14^. Two seminal papers have recently shown that the addition of JAK inhibitors to immunotherapy in vivo enhances the anti-tumour response to immunotherapy^30,31^. This was then replicated clinically in a small cohort of patients with non-Hodgkin’s lymphoma and non-small cell lung cancer, whereby patients received immunotherapy in combination with either ruxolitinib or itactinib. Both studies illustrated promising response rates and PFS. When considering the subgroup of participants that had received the atezolizumab/bevacizumab first-line in this trial, a significantly higher risk of disease progression was observed following itacitinib when compared to those who received TKIs first line. The difference between our results and the recent combinatorial publications may be due to the timing and regimen of immunotherapy used. Zak and Mathews and colleagues used JAK inhibitors in combination with immunotherapy, whilst the subgroup in our study received sequential therapy. Sequential, sustained therapy with itacitinib may result in blockade of anti-tumour activity induced by cytokines, limiting lymphocyte activation and proliferation. Whilst our study was not powered to address the issue of combination immunotherapy and JAK inhibition, these results suggest that careful timing needs to be explored in future studies, underpinned by translational endpoints.

We undertook targeted immune profiling of tissue samples prior to itacitinib receipt. In responders, there was a clear upregulation of genes responsible for a pro-inflammatory response, including JAK1, TGFR-β and IL-1R1, whilst there was downregulation in RNA expression of phospholipase A2, PSMB8, TAP1 and the chemoattractant CXCL11. The upregulation of JAK1 and TGFR-β combined with IL-1R1 is suggestive of a sustained pro-inflammatory response^32,33^. The reduction of phospholipase A2 gene expression is consistent with activation of JAK, which reduces the production of bioactive phospholipids, including the eicosanoids and lysophosphatidic acid, impacting on inflammation and immunity^34^. PSMB8 forms part of the immunoproteosome complex, which is involved in antigen presentation through MHC class I, whilst TAP1 regulates antigen presentation by MHC class I and subsequent recruitment of regulatory T cells^35–37^. Taken together, these findings are supported by previous work suggesting uncontrolled inflammation driven by IL-6 promotes immune suppression through reduced antigen presentation and chemotactic recruitment^38^. This phenotype is suggestive of a dual strategy by the tumour of potentiating tumour-relevant inflammation whilst inhibiting adaptive immune surveillance. It can be hypothesised that, as a specific inhibitor of JAK1, itacitinib potently inhibits the phosphorylation of STAT proteins and production of pro-inflammatory cytokines, restoring the immune microenvironment and improving outcomes.

Considering the RNA sequencing results, we then undertook an untargeted metabonomics approach to ascertain if a downstream molecular signature predicted for clinical outcome to itacitinib. We identified a unique metabolic signature in serum that accurately predicts both response and survival. We observed robust differences in two small molecules and nine lipid species, namely treatment, an increase in acetic acid and proline and a reduction in the relative abundance of L1AB, L1CH, L1FC, L1PL, L1PN, L3AB, L3PN, TBPN, and TPAB. Collectively, this panel of 11 metabolites gave a high predictive accuracy of clinical outcome. In particular, elevated levels of acetic acid and proline predicted for response and improved survival. The panel identified may either suggest a pro-inflammatory state receptive to itacitinib therapy or a metabolite signature predictive of a phenotype with a good prognosis, the latter being suggested by raised levels of acetic acid.

Acetic acid is the main short-chain fatty acid (SCFA) produced by fermentation of non-digestible carbohydrates, proteins, and other substrates in the colon by bacterial species^39^. Unlike other SCFAs, acetic acid is the only SCFA to reach the systemic circulation, where the acetyl group is metabolised to acetate, a substrate of acetyl-CoA and the TCA cycle, fundamental in supporting lipogenesis and protein acetylation, processes that are increased during carcinogenesis. There is a significant body of literature describing changes in the gut microbiome and its impact on systemic therapy, with studies illustrating that response to treatment can be predicted from the microbiome^40^. Enrichment of certain bacterial species, such as *Faecalibacterium*, *Bifidobacterium*, *Lactobacillus*, *Akkermansia muciniphila*, and *Ruminococcaceae* spp. has been found to have a favourable impact on immunotherapy response, whilst dysbiosis is associated with a shift towards aerobes and decreased levels of SCFAs, which in turn reduces the immune response, suggesting that acetic acid may act as a predictive biomarker of response^41,42^. Within the tumour microenvironment, SCFAs enable the differentiation of regulatory T cells and T helper cells, facilitating an immune-responsive environment, with numerous studies illustrating an anti-inflammatory effect of SCFAs and improved outcomes^43,44^. Future work should consider the examination of the faecal microbiome during itacitinib therapy.

The role of proline metabolism as a regulator of tumour growth and metastasis is increasingly being recognised^45^ Proline is a key component of collagen, and increased levels are observed in states of disease where there is an increased demand for collagen synthesis, including fibrosis, as in cirrhosis, and the creation of the extracellular matrix associated with malignancy^46^. Proline metabolism regulates the redox pathway, protecting cells from raised intracellular reactive oxygen species levels in response to TGFβ^47^. A key enzyme in proline synthesis is PYCR1. PYCR1 is one of the most commonly overexpressed metabolic genes in cancer, including HCC, where knockdown of PYCR1 suppresses tumour growth and proliferation in vitro^45,48^. Moreover, increased expression of *PYCR1* has been shown to be associated with resistance to immunotherapy and increased infiltration of immunosuppressive myeloid-derived suppressor cells, regulatory T cells, and tumour-infiltrating macrophages^49^. As *PYCRI* binds to STAT3, promoting signalling, it can be postulated that the improved outcome for patients with elevated proline may be related to repression of the inflammatory signalling with itacitinib, which in turn reduces proline biosynthesis; however, this hypothesis requires further investigation^50^.

With regard to the lipid classes contributing to clinical outcome, we observed that a relative reduction in multiple LDL species and the main constituent of LDL, apolipoprotein B. Circulating LDL binds to the LDL-receptors, 70% of which are expressed in the liver, facilitating the removal of LDL from the systemic circulation. The expression of LDL-R is regulated by the master nuclear receptor, LXR. There is an increasing body of evidence to suggest that LXR acts in an anti-inflammatory manner, repressing pro-inflammatory gene expression, and the predictive lipid phenotype observed may reflect patients with an established pro-inflammatory state who have an improved response to itacitinib^51^. Decreases in lipid species may also reflect increased lipid catabolism through β-oxidation or membrane remodelling in responders, and further investigation may include integrating both genomics and metabonomics in a larger sample size to delineate these hypotheses further^52,53^. Taken together, our circulating findings suggest that the presence of a pro-inflammatory state predicts for clinical response to itacitinib; however, these findings need to be further investigated in larger, prospective trials.

There are a number of key limitations which need to be considered in interpreting the results. The sample size is limited, and there was a lack of a control arm. These preliminary findings do require further evaluation in a randomised phase II study to be able to draw definitive conclusions. The COVID-19 pandemic resulted in a pause in clinical trial activities during which new first and second-line therapies became available, which resulted in changes to the original protocol, and significant delays in participant recruitment, which may have impacted the clinical outcomes. The pandemic also resulted in a reduction in the number of samples available for translational analyses. The pandemic also resulted in *Future work should involve a larger, phase II study comparing itacitinib with TKI in the second line setting supported by mechanistic work which should include analysis of circulating cytokines and changes with itacitinib therapy as well consideration of mutational analysis on both cfDNA and tumour biopsies to assess whether mutations in JAK1 may be predictive of outcome. JAK1* is mutated in 10% of HCC and may result in hyperactivation of the JAK/STAT pathway^54^. A study investigating *JAK1*^*S703I*^ mutations in HCC cell lines demonstrated increased sensitivity to ruxolitinib in mutated cell lines when compared to wild-type and could be used as a potential biomarker for itacitinib response^55^. In conclusion, through a phase Ib study, we have shown that inhibition of JAK1 signalling using itacitinib was associated with improved PFS in line with previous studies in the second/third line setting, albeit in a heavily pretreated cohort with underlying liver dysfunction. We have identified a pretreatment inflammatory signal predictive of response that, whilst provocative, requires further evaluation in larger studies of JAK inhibitors in combination with immunotherapy.

## Methods

### Study design and eligibility

JAKaL was a phase 1b, open-label, single-arm study of itacitinib in treatment-experienced patients with advanced/metastatic HCC. The study protocol has been previously published^56^. The study was conducted at Hammersmith Hospital, Imperial College Healthcare NHS Trust. Eligible patients were aged ≥18 years, had a confirmed radiologic or histologic diagnosis of HCC based on American Association of Study of Liver Diseases criteria, Child-Pugh cirrhosis score <B8, progression or intolerance to at least one line of therapy and undetectable or low HBV/HCV viral load^57^.

The study was conducted in accordance with Good Clinical Practice standards and in respect of the ethical framework stipulated within the Declaration of Helsinki. The study was approved by the Yorkshire & The Humber - Sheffield Research Ethics Committee and Health Regulator Authority; EudraCT number: 2017-004437-81. ClinicalTrials.gov identifier: NCT04358185. All patients provided written informed consent prior to participation.

### Study procedures

Following consent and screening, patients commenced itacitinib 400 mg orally once daily (QD) for a 28-day cycle. AEs were assessed at baseline, then weekly for the first 28 days and every 4 weeks thereafter. Tumour assessment was done by staging CT scans of the chest, abdomen and pelvis were performed at baseline, then every 8 weeks from treatment initiation until treatment cessation. All participants remained on treatment until confirmed disease progression, unacceptable toxicities, withdrawal of consent, investigator decision or death by any cause, whichever occurred first.

### Outcomes

The primary endpoint of the study was the safety and tolerability of itacitinib. All participants who received at least one dose of itacitinib were included in the safety analysis. The causality between AEs and treatment was assigned by the investigators. Patients were assessed for the emergence of AEs up to 30 days after the end of treatment (EOT). A DLT was defined as a treatment-related ≥grade 3 AE occurring during the assessment window of 28 days from the first administration of itacitinib.

In case of toxicity, participants could interrupt the trial drug if deemed appropriate by the team, for a maximum of 14 days. For any DLT, defined as any Grade 3 or higher toxicity resulting in a dose reduction, the following de-escalation and re-escalation pathway was applied:

- For grade 3 toxicity, itacitinib was held drug until toxicity resolved to grade ≤1, then resumed at 400 mg QD. If the participant experienced a second grade 3 toxicity, itacitinib was withheld until resolution of toxicity to grade ≤1, then resumed at 300 mg QD. If toxicity remained grade ≤1 after 28 days, the drug would be re-escalated to 400 mg QD.
- If grade 4 toxicity was experienced, itacitinib was withheld until toxicity resolved to grade ≤1, then resumed at a reduced dose level of 300 mg QD or discontinued at the discretion of the investigator.

Any toxicity or AE were assessed at each visit at the beginning of the cycle and at the EOT. Safety assessments included physical and laboratory findings, and trAEs were defined according to the National Cancer Institute (NCI) Common Toxicity Criteria for Adverse Events version 4.03.

Data on response and survival were collected and analysed on an intention-to-treat basis. Response data was considered evaluable only if participants had at least one follow-up scan following treatment initiation. Response was assessed according to RECIST 1.1 criteria^58^.

PFS was defined by the time from first commencement of itacitinib to the first occurrence of documented disease progression, based on RECIST 1.1, or death from any cause, whichever occurred first. OS was defined from the time of screening visit till death from any cause. PFS was censored at the time of last tumour assessment in those patients who had not progressed by the time of database lock (30/4/2023), whereas OS was censored at the time of last patient contact. Survival follow-up was carried out every 8 weeks after the EOT.

### Translational endpoints

Optional tumour biopsies were collected at screening. Two cores were collected from each biopsy: one of which was snap frozen and the other paraffin-embedded.

Blood samples were collected at the screening visit, the beginning of each treatment cycle and EOT. Serum and plasma were collected, and all samples were stored at −80 °C until analysed.

### Targeted transcriptomics

Tumour biopsies performed prior to commencing therapy were reviewed for the presence of an adequate percentage of cancer cells by a consultant histopathologist (RG). RNA was purified from samples using the Allprep DNA/RNA mini kit (Qiagen, Venlo, NL, Cat. 80234). RNA quantification and quality control were performed using the ND2000 Nanodrop spectrophotometer (Thermo Fisher Scientific, Loughborough, UK). Isolated RNA underwent targeted sequencing using NanoString Human Immunology V2 panel of 579 genes (Supplementary Note 1) (NanoString Technologies, Seattle, USA). Quality control and transcriptomic analysis were performed using the nCounter® Analysis System (NanoString Technologies, Seattle, USA). Differential gene expression profiles were calculated between responders and non-responders. GSEA was performed using 32 gene expression profiles (Supplementary Note 2).

### Serum metabolomic profiling

Metabolomic analyses of serum samples were performed by ^1^H-nuclear magnetic resonance spectroscopy (^1^H-NMR) using a Bruker Advance III HD 600 spectrometer (Billerica, Massachusetts, USA) using previously published standard protocols^59^. One-dimensional ^1^H-NMR general profile (Noesy and CPMG) and two-dimensional J-resolved experiments were acquired, as previously described^59^. Experiments were acquired and processed in automation using TopSpin 3.6.2 and ICON NMR. Phasing, baseline correction and calibration to TSP were also carried out in automation after each acquisition. Spectra quality was assessed using an in-house developed bioinformatics tool nPYc^59,60^. The full list of serum metabolites analysed is reported in Supplementary Note 3.

### Signature discovery using partial least squares regression

Raw metabolite abundance data were processed using the *MetaboAnalystR (v4.0.0)* package. There were no missing values in raw abundance data. Median centring was performed for row-wise normalisation, followed by log-transformation to reduce skewness in data distribution, and lastly mean-centring scaling to standardise the data for downstream statistical analysis using the *Normalization* function with the following parameters: *rownorm* = ‘*MedianNorm’, transNorm* = ‘*LogNorm’, scaleNorm* = ‘*MeanCenter’*, ratio = FALSE. The effect of normalisation was visually assessed using the panel of box plots and density plots.

Multivariate analysis, including principal component analysis (PCA) and partial least squares-discriminant analysis (PLS-DA), was performed to explore patterns and group separations within the data. PCA was conducted using the *PCA.Anal* function, while PLS-DA was performed with the *PLSR.Anal* function. Cross-validation for PLS-DA was carried out using the PLSDA.CV function with the following parameters: *cvOpt* = ‘*LOOCV*’*, compNum* = *5, and choice* = ‘*Q2*’. The analysis provided VIP scores, which indicate how much each metabolite contributes to distinguishing between groups. Higher VIP scores mean greater importance. Additionally, PLS-DA permutation testing (1000 permutations) was done using the *PLSDA.Permut* function, with empirical p-values indicating the likelihood that the classification result occurred by chance.

Following the PLS-DA analysis, ROC curve analysis was performed to assess the classification performance of the model. The *PrepareROCData* function was used to prepare the data for ROC analysis, and cross-validation was conducted using the *PerformCV.explore* function with the classification method (*cls.method*) set to ‘PLS’ and the ranking method (*rank.method*) also set to ‘PLS’, specifying *lvNum* = *2* to limit the model to two latent variables. The ROC curves were generated using the *PlotROC* function, where *avg.method* = ‘*threshold’* was applied to calculate the ROC curve based on threshold averaging, and confidence intervals were displayed by setting *show.conf* = *1*. Predicted class probabilities were visualised using the *PlotProbView* function, and model accuracy was assessed with the *PlotAccuracy* function. The predicted score for each sample was calculated by averaging the predicted class probabilities across components. The statistical significance of the differences in predicted score distributions between the two groups, complete response and partial response, was evaluated using the Wilcoxon rank-sum test.

### Inter-metabolite correlation

Heatmaps were generated using Pearson correlation coefficients to show correlations between features, created by the *pheatmap (v1.0.12)* package. Hierarchical clustering was applied to group metabolites with similar patterns across samples, and dendrograms were used to visualise these clusters. The correlations were colour-coded, with pink indicating values close to 1, blue for values near −1, and white for correlations around 0.

### Statistics

All patients who received at least one cycle of itacitinib were included in the safety and efficacy analysis. Demographics, safety and efficacy data were analysed using descriptive statistical measures. OS and PFS were analysed using Kaplan–Meier statistics, and Cox-regression analysis was performed on the same data sets.

Responders were classified as those experiencing CR, PR and SD, and non-responders were those experiencing PD as the best overall response by RECIST v1.1. The software used for the data analysis was SPSS v28. Translational data were analysed and plotted using GraphPad Prism v9.5.

**Fig. 7:**
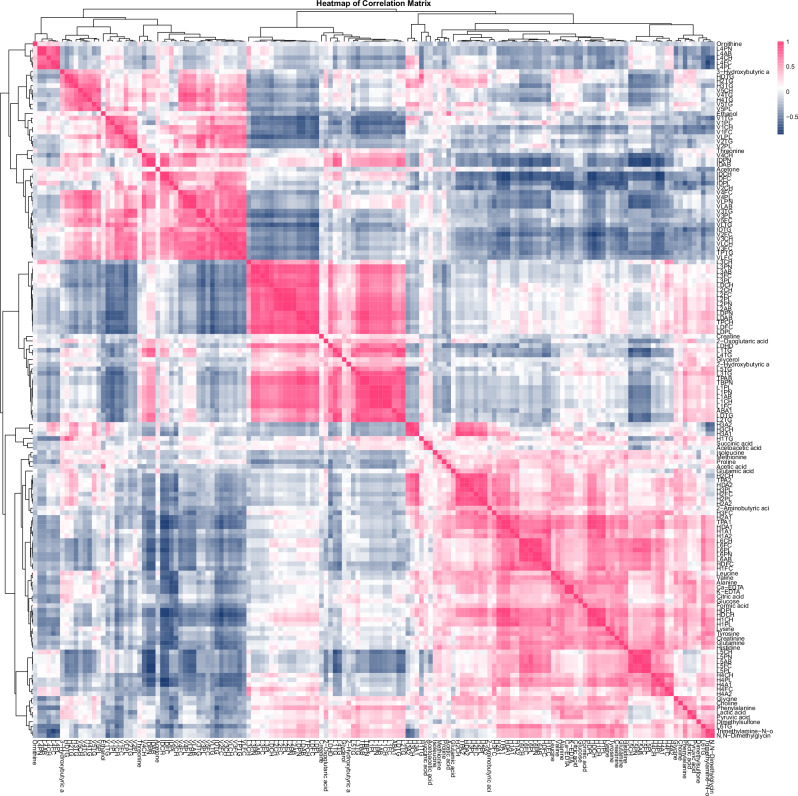
Spearman correlation matrix of metabolite intensities. Heatmap displaying pairwise Spearman’s rank correlation coefficients (*ρ*) of metabolite intensities across all samples. Rows and columns were hierarchically clustered using Euclidean distance. The colour scale represents correlation strength: blue (negative correlation; −1 ≤ *ρ* < 0), white (*ρ* = 0; no correlation), and red (positive correlation; 0 < *ρ* ≤ 1).

For metabolite analysis, Kaplan–Meier survival curves for OS and PFS were generated using the *ggsurvplot* function from the *survminer (v0.4.9)* package. Patients were divided into high or low abundance groups based on whether their metabolite levels were above or below the average for each specific metabolite. Hazard ratios and log-rank p-values were calculated using the *coxph* function from *survival (v3.7-0)* package to evaluate statistical significance (Fig. 7).

## Supplementary information

Supplementary

## Data Availability

Data will be made available on request.
