# Supplementary material for: Itacitinib in advanced hepatocellular cancer following first line therapy
Source: NPJ Precis Oncol. 2026 Feb 11;10:67. doi: 10.1038/s41698-026-01273-9 (PMC12905154; doi:10.1038/s41698-026-01273-9)

**Supplementary Note 1. – Targeted genes from gene panel using NanoString Human Immunology panel.**

| **Gene Name** | **Official Full Name** |
| --- | --- |
| ABCB1 | ATP-binding cassette, sub-family B (MDR/TAP), member 1 |
| ABL1 | c-abl oncogene 1, non-receptor tyrosine kinase |
| ADA | adenosine deaminase |
| AHR | aryl hydrocarbon receptor |
| AICDA | activation-induced cytidine deaminase |
| AIRE | autoimmune regulator |
| APP | amyloid beta (A4) precursor protein |
| ARG1 | arginase, liver |
| ARG2 | arginase, type II |
| ARHGDIB | Rho GDP dissociation inhibitor (GDI) beta |
| ATG10 | ATG10 autophagy related 10 homolog (S. cerevisiae) |
| ATG12 | ATG12 autophagy related 12 homolog (S. cerevisiae) |
| ATG16L1 | ATG16 autophagy related 16-like 1 (S. cerevisiae) |
| ATG5 | ATG5 autophagy related 5 homolog (S. cerevisiae) |
| ATG7 | ATG7 autophagy related 7 homolog (S. cerevisiae) |
| ATM | ataxia telangiectasia mutated |
| B2M | beta-2-microglobulin |
| B3GAT1 | beta-1,3-glucuronyltransferase 1 (glucuronosyltransferase P) |
| BATF | basic leucine zipper transcription factor, ATF-like |
| BATF3 | basic leucine zipper transcription factor, ATF-like 3 |
| BAX | BCL2-associated X protein |
| BCAP31 | B-cell receptor-associated protein 31 |
| BCL10 | B-cell CLL/lymphoma 10 |
| BCL2 | B-cell CLL/lymphoma 2 |
| BCL2L11 | BCL2-like 11 (apoptosis facilitator) |
| BCL3 | B-cell CLL/lymphoma 3 |
| BCL6 | B-cell CLL/lymphoma 6 |
| BID | BH3 interacting domain death agonist |
| BLNK | B-cell linker |
| BST1 | bone marrow stromal cell antigen 1 |
| BST2 | bone marrow stromal cell antigen 2 |
| BTK | Bruton agammaglobulinemia tyrosine kinase |
| BTLA | B and T lymphocyte associated |
| C14orf166 | chromosome 14 open reading frame 166 |
| C1QA | complement component 1, q subcomponent, A chain |
| C1QB | complement component 1, q subcomponent, B chain |
| C1QBP | complement component 1, q subcomponent binding protein |
| C1R | complement component 1, r subcomponent |
| C1S | complement component 1, s subcomponent |
| C2 | complement component 2 |
| C3 | complement component 3 |
| C4A/B | complement component 4A (Rodgers blood group)/complement component 4B (Chido blood group) |
| C4BPA | complement component 4 binding protein, alpha |
| C5 | complement component 5 |
| C6 | complement component 6 |
| C7 | complement component 7 |
| C8A | complement component 8, alpha polypeptide |
| C8B | complement component 8, beta polypeptide |
| C8G | complement component 8, gamma polypeptide |
| C9 | complement component 9 |
| CAMP | cathelicidin antimicrobial peptide |
| CARD9 | caspase recruitment domain family, member 9 |
| CASP1 | caspase 1, apoptosis-related cysteine peptidase (interleukin 1, beta, convertase) |
| CASP10 | caspase 10, apoptosis-related cysteine peptidase |
| CASP2 | caspase 2, apoptosis-related cysteine peptidase |
| CASP3 | caspase 3, apoptosis-related cysteine peptidase |
| CASP8 | caspase 8, apoptosis-related cysteine peptidase |
| CCBP2 | chemokine binding protein 2 |
| CCL11 | chemokine (C-C motif) ligand 11 |
| CCL13 | chemokine (C-C motif) ligand 13 |
| CCL15 | chemokine (C-C motif) ligand 15 |
| CCL16 | chemokine (C-C motif) ligand 16 |
| CCL18 | chemokine (C-C motif) ligand 18 (pulmonary and activation-regulated) |
| CCL19 | chemokine (C-C motif) ligand 19 |
| CCL2 | chemokine (C-C motif) ligand 2 |
| CCL20 | chemokine (C-C motif) ligand 20 |
| CCL22 | chemokine (C-C motif) ligand 22 |
| CCL23 | chemokine (C-C motif) ligand 23 |
| CCL24 | chemokine (C-C motif) ligand 24 |
| CCL26 | chemokine (C-C motif) ligand 26 |
| CCL3 | chemokine (C-C motif) ligand 3 |
| CCL4 | chemokine (C-C motif) ligand 4 |
| CCL5 | chemokine (C-C motif) ligand 5 |
| CCL7 | chemokine (C-C motif) ligand 7 |
| CCL8 | chemokine (C-C motif) ligand 8 |
| CCND3 | cyclin D3 |
| CCR1 | chemokine (C-C motif) receptor 1 |
| CCR10 | chemokine (C-C motif) receptor 10 |
| CCR2 | chemokine (C-C motif) receptor 2 |
| CCR5 | chemokine (C-C motif) receptor 5 |
| CCR6 | chemokine (C-C motif) receptor 6 |
| CCR7 | chemokine (C-C motif) receptor 7 |
| CCR8 | chemokine (C-C motif) receptor 8 |
| CCRL1 | chemokine (C-C motif) receptor-like 1 |
| CCRL2 | chemokine (C-C motif) receptor-like 2 |
| CD14 | CD14 molecule |
| CD160 | CD160 molecule |
| CD163 | CD163 molecule |
| CD164 | CD164 molecule, sialomucin |
| CD19 | CD19 molecule |
| CD1A | CD1a molecule |
| CD1D | CD1d molecule |
| CD2 | CD2 molecule |
| CD209 | CD209 molecule |
| CD22 | CD22 molecule |
| CD24 | CD24 molecule |
| CD244 | CD244 molecule, natural killer cell receptor 2B4 |
| CD247 | CD247 molecule |
| CD27 | CD27 molecule |
| CD274 | CD274 molecule |
|  |  |
| CD276 | CD276 molecule |
| CD28 | CD28 molecule |
| CD34 | CD34 molecule |
| CD36 | CD36 molecule (thrombospondin receptor) |
| CD3D | CD3d molecule, delta (CD3-TCR complex) |
| CD3E | CD3e molecule, epsilon (CD3-TCR complex) |
| CD3EAP | CD3e molecule, epsilon associated protein |
| CD4 | CD4 molecule |
| CD40 | CD40 molecule, TNF receptor superfamily member 5 |
| CD40LG | CD40 ligand |
| CD44 | CD44 molecule (Indian blood group) |
| CD46 | CD46 molecule, complement regulatory protein |
| CD48 | CD48 molecule |
| CD5 | CD5 molecule |
| CD53 | CD53 molecule |
| CD55 | CD55 molecule, decay accelerating factor for complement (Cromer blood group) |
| CD58 | CD58 molecule |
| CD59 | CD59 molecule, complement regulatory protein |
| CD6 | CD6 molecule |
| CD7 | CD7 molecule |
| CD70 | CD70 molecule |
| CD74 | CD74 molecule, major histocompatibility complex, class II invariant chain |
| CD79A | CD79a molecule, immunoglobulin-associated alpha |
| CD79B | CD79b molecule, immunoglobulin-associated beta |
| CD80 | CD80 molecule |
| CD81 | CD81 molecule |
| CD82 | CD82 molecule |
| CD83 | CD83 molecule |
| CD86 | CD86 molecule |
| CD8A | CD8a molecule |
| CD8B | CD8b molecule |
| CD9 | CD9 molecule |
| CD96 | CD96 molecule |
| CD97 | CD97 molecule |
| CD99 | CD99 molecule |
| CDH5 | cadherin 5, type 2 (vascular endothelium) |
| CDKN1A | cyclin-dependent kinase inhibitor 1A (p21, Cip1) |
| CEACAM1 | carcinoembryonic antigen-related cell adhesion molecule 1 (biliary glycoprotein) |
| CEACAM6 | carcinoembryonic antigen-related cell adhesion molecule 6 (non-specific cross reacting antigen) |
| CEACAM8 | carcinoembryonic antigen-related cell adhesion molecule 8 |
| CEBPB | CCAAT/enhancer binding protein (C/EBP), beta |
| CFB | complement factor B |
| CFD | complement factor D (adipsin) |
| CFH | complement factor H |
| CFI | complement factor I |
| CFP | complement factor properdin |
| CHUK | conserved helix-loop-helix ubiquitous kinase |
| CIITA | class II, major histocompatibility complex, transactivator |
| CISH | cytokine inducible SH2-containing protein |
| CLEC4A | C-type lectin domain family 4, member A |
| CLEC4E | C-type lectin domain family 4, member E |
| CLEC5A | C-type lectin domain family 5, member A |
| CLEC6A | C-type lectin domain family 6, member A |
| CLEC7A | C-type lectin domain family 7, member A |
| CLU | clusterin |
| CMKLR1 | chemokine-like receptor 1 |
| CR1 | complement component (3b/4b) receptor 1 (Knops blood group) |
| CR2 | complement component (3d/Epstein Barr virus) receptor 2 |
| CRADD | CASP2 and RIPK1 domain containing adaptor with death domain |
| CSF1 | colony stimulating factor 1 (macrophage) |
| CSF1R | colony stimulating factor 1 receptor |
| CSF2 | colony stimulating factor 2 (granulocyte-macrophage) |
| CSF2RB | colony stimulating factor 2 receptor, beta, low-affinity (granulocyte-macrophage) |
| CSF3R | colony stimulating factor 3 receptor (granulocyte) |
| CTLA4_all | cytotoxic T-lymphocyte-associated protein 4 |
| CTLA4-TM | cytotoxic T-lymphocyte-associated protein 4 |
| sCTLA4 | cytotoxic T-lymphocyte-associated protein 4 |
| CTNNB1 | catenin (cadherin-associated protein), beta 1, 88kDa |
| CTSC | cathepsin C |
| CTSG | cathepsin G |
| CTSS | cathepsin S |
| CUL9 | cullin 9 |
| CX3CL1 | chemokine (C-X3-C motif) ligand 1 |
| CX3CR1 | chemokine (C-X3-C motif) receptor 1 |
| CXCL1 | chemokine (C-X-C motif) ligand 1 (melanoma growth stimulating activity, alpha) |
| CXCL10 | chemokine (C-X-C motif) ligand 10 |
| CXCL11 | chemokine (C-X-C motif) ligand 11 |
| CXCL12 | chemokine (C-X-C motif) ligand 12 |
| CXCL13 | chemokine (C-X-C motif) ligand 13 |
| CXCL2 | chemokine (C-X-C motif) ligand 2 |
| CXCL9 | chemokine (C-X-C motif) ligand 9 |
| CXCR1 | chemokine (C-X-C motif) receptor 1 |
| CXCR2 | chemokine (C-X-C motif) receptor 2 |
| CXCR3 | chemokine (C-X-C motif) receptor 3 |
| CXCR4 | chemokine (C-X-C motif) receptor 4 |
| CXCR6 | chemokine (C-X-C motif) receptor 6 |
| CYBB | cytochrome b-245, beta polypeptide |
| DEFB1 | defensin, beta 1 |
| DEFB103A | defensin, beta 103A |
| DEFB103B | defensin, beta 103B |
| DEFB4A | defensin, beta 4A |
| DPP4 | dipeptidyl-peptidase 4 |
| DUSP4 | dual specificity phosphatase 4 |
| EBI3 | Epstein-Barr virus induced 3 |
| EDNRB | endothelin receptor type B |
| EGR1 | early growth response 1 |
| EGR2 | early growth response 2 |
| ENTPD1 | ectonucleoside triphosphate diphosphohydrolase 1 |
| EOMES | eomesodermin |
| ETS1 | v-ets erythroblastosis virus E26 oncogene homolog 1 (avian) |
| FADD | Fas (TNFRSF6)-associated via death domain |
| FAS | Fas (TNF receptor superfamily, member 6) |
| FCAR | Fc fragment of IgA, receptor for |
| FCER1A | Fc fragment of IgE, high affinity I, receptor for; alpha polypeptide |
| FCER1G | Fc fragment of IgE, high affinity I, receptor for; gamma polypeptide |
| FCGR1A/B | Fc fragment of IgG, high affinity Ia, receptor (CD64)/Fc fragment of IgG, high affinity Ib, receptor (CD64) |
| FCGR2A | Fc fragment of IgG, low affinity IIa, receptor (CD32) |
| FCGR2A/C | Fc fragment of IgG, low affinity IIa, receptor (CD32)/Fc fragment of IgG, low affinity IIc, receptor for (CD32) |
| FCGR2B | Fc fragment of IgG, low affinity IIb, receptor (CD32) |
| FCGR3A/B | Fc fragment of IgG, low affinity IIIa, receptor (CD16a)/Fc fragment of IgG, low affinity IIIb, receptor (CD16a) |
| FCGRT | Fc fragment of IgG, receptor, transporter, alpha |
| FKBP5 | FK506 binding protein 5 |
| FN1 | fibronectin 1 |
| FOXP3 | forkhead box P3 |
| FYN | FYN oncogene related to SRC, FGR, YES |
| GATA3 | GATA binding protein 3 |
| GBP1 | guanylate binding protein 1, interferon-inducible |
| GBP5 | guanylate binding protein 5 |
| GFI1 | growth factor independent 1 transcription repressor |
| GNLY | granulysin |
| GP1BB | glycoprotein Ib (platelet), beta polypeptide |
| GPI | glucose-6-phosphate isomerase |
| GPR183 | G protein-coupled receptor 183 |
| GZMA | granzyme A (granzyme 1, cytotoxic T-lymphocyte-associated serine esterase 3) |
| GZMB | granzyme B (granzyme 2, cytotoxic T-lymphocyte-associated serine esterase 1) |
| GZMK | granzyme K (granzyme 3; tryptase II) |
| HAMP | hepcidin antimicrobial peptide |
| HAVCR2 | hepatitis A virus cellular receptor 2 |
| HFE | hemochromatosis |
| HLA-A | major histocompatibility complex, class I, A |
| HLA-B | major histocompatibility complex, class I, B |
| HLA-C | major histocompatibility complex, class I, C |
| HLA-DMA | major histocompatibility complex, class II, DM alpha |
| HLA-DMB | major histocompatibility complex, class II, DM beta |
| HLA-DOB | major histocompatibility complex, class II, DO beta |
| HLA-DPA1 | major histocompatibility complex, class II, DP alpha 1 |
| HLA-DPB1 | major histocompatibility complex, class II, DP beta 1 |
| HLA-DQA1 | major histocompatibility complex, class II, DQ alpha 1 |
| HLA-DQB1 | major histocompatibility complex, class II, DQ beta 1 |
| HLA-DRA | major histocompatibility complex, class II, DR alpha |
| HLA-DRB1 | major histocompatibility complex, class II, DR beta 1 |
| HLA-DRB3 | major histocompatibility complex, class II, DR beta 3 |
| HRAS | v-Ha-ras Harvey rat sarcoma viral oncogene homolog |
| ICAM1 | intercellular adhesion molecule 1 |
| ICAM2 | intercellular adhesion molecule 2 |
| ICAM3 | intercellular adhesion molecule 3 |
| ICAM4 | intercellular adhesion molecule 4 (Landsteiner-Wiener blood group) |
| ICAM5 | intercellular adhesion molecule 5, telencephalin |
| ICOS | inducible T-cell co-stimulator |
| ICOSLG | inducible T-cell co-stimulator ligand |
| IDO1 | indoleamine 2,3-dioxygenase 1 |
| IFI16 | interferon, gamma-inducible protein 16 |
| IFI35 | interferon-induced protein 35 |
| IFIH1 | interferon induced with helicase C domain 1 |
| IFIT2 | interferon-induced protein with tetratricopeptide repeats 2 |
| IFITM1 | interferon induced transmembrane protein 1 (9-27) |
| IFNA1/13 | interferon, alpha 1/interferon, alpha 13 |
| IFNA2 | interferon, alpha 2 |
| IFNAR1 | interferon (alpha, beta and omega) receptor 1 |
| IFNAR2 | interferon (alpha, beta and omega) receptor 2 |
| IFNB1 | interferon, beta 1, fibroblast |
| IFNG | interferon, gamma |
| IFNGR1 | interferon gamma receptor 1 |
| IGF2R | insulin-like growth factor 2 receptor |
| IKBKAP | inhibitor of kappa light polypeptide gene enhancer in B-cells, kinase complex-associated protein |
| IKBKB | inhibitor of kappa light polypeptide gene enhancer in B-cells, kinase beta |
| IKBKE | inhibitor of kappa light polypeptide gene enhancer in B-cells, kinase epsilon |
| IKBKG | inhibitor of kappa light polypeptide gene enhancer in B-cells, kinase gamma |
| IKZF1 | IKAROS family zinc finger 1 (Ikaros) |
| IKZF2 | IKAROS family zinc finger 2 (Helios) |
| IKZF3 | IKAROS family zinc finger 3 (Aiolos) |
| IL10 | interleukin 10 |
| IL10RA | interleukin 10 receptor, alpha |
| IL11RA | interleukin 11 receptor, alpha |
| IL12A | interleukin 12A (natural killer cell stimulatory factor 1, cytotoxic lymphocyte maturation factor 1, p35) |
| IL12B | interleukin 12B (natural killer cell stimulatory factor 2, cytotoxic lymphocyte maturation factor 2, p40) |
| IL12RB1 | interleukin 12 receptor, beta 1 |
| IL13 | interleukin 13 |
| IL13RA1 | interleukin 13 receptor, alpha 1 |
| IL15 | interleukin 15 |
| IL16 | interleukin 16 |
| IL17A | interleukin 17A |
| IL17B | interleukin 17B |
| IL17F | interleukin 17F |
| IL18 | interleukin 18 (interferon-gamma-inducing factor) |
| IL18R1 | interleukin 18 receptor 1 |
| IL18RAP | interleukin 18 receptor accessory protein |
| IL19 | interleukin 19 |
| IL1A | interleukin 1, alpha |
| IL1B | interleukin 1, beta |
| IL1R1 | interleukin 1 receptor, type I |
| IL1R2 | interleukin 1 receptor, type II |
| IL1RAP | interleukin 1 receptor accessory protein |
| IL1RL1 | interleukin 1 receptor-like 1 |
| IL1RL2 | interleukin 1 receptor-like 2 |
| IL1RN | interleukin 1 receptor antagonist |
| IL2 | interleukin 2 |
| IL20 | interleukin 20 |
| IL21 | interleukin 21 |
| IL21R | interleukin 21 receptor |
| IL22 | interleukin 22 |
| IL22RA2 | interleukin 22 receptor, alpha 2 |
| IL23A | interleukin 23, alpha subunit p19 |
| IL23R | interleukin 23 receptor |
| IL26 | interleukin 26 |
| IL27 | interleukin 27 |
| IL28A | interleukin 28A (interferon, lambda 2) |
| IL28A/B | interleukin 28A (interferon, lambda 2)/interleukin 28B (interferon, lambda 3) |
| IL29 | interleukin 29 (interferon, lambda 1) |
| IL2RA | interleukin 2 receptor, alpha |
| IL2RB | interleukin 2 receptor, beta |
| IL2RG | interleukin 2 receptor, gamma |
| IL3 | interleukin 3 (colony-stimulating factor, multiple) |
| IL32 | interleukin 32 |
| IL4 | interleukin 4 |
| IL4R | interleukin 4 receptor |
| IL5 | interleukin 5 (colony-stimulating factor, eosinophil) |
| IL6 | interleukin 6 (interferon, beta 2) |
| IL6R | interleukin 6 receptor |
| IL6ST | interleukin 6 signal transducer (gp130, oncostatin M receptor) |
| IL7 | interleukin 7 |
| IL7R | interleukin 7 receptor |
| IL8 | interleukin 8 |
| IL9 | interleukin 9 |
| ILF3 | interleukin enhancer binding factor 3, 90kDa |
| IRAK1 | interleukin-1 receptor-associated kinase 1 |
| IRAK2 | interleukin-1 receptor-associated kinase 2 |
| IRAK3 | interleukin-1 receptor-associated kinase 3 |
| IRAK4 | interleukin-1 receptor-associated kinase 4 |
| IRF1 | interferon regulatory factor 1 |
| IRF3 | interferon regulatory factor 3 |
| IRF4 | interferon regulatory factor 4 |
| IRF5 | interferon regulatory factor 5 |
| IRF7 | interferon regulatory factor 7 |
| IRF8 | interferon regulatory factor 8 |
| IRGM | immunity-related GTPase family, M |
| ITGA2B | integrin, alpha 2b (platelet glycoprotein IIb of IIb/IIIa complex, antigen CD41) |
| ITGA4 | integrin, alpha 4 (antigen CD49D, alpha 4 subunit of VLA-4 receptor) |
| ITGA5 | integrin, alpha 5 (fibronectin receptor, alpha polypeptide) |
| ITGA6 | integrin, alpha 6 |
| ITGAE | integrin, alpha E (antigen CD103, human mucosal lymphocyte antigen 1; alpha polypeptide) |
| ITGAL | integrin, alpha L (antigen CD11A (p180), lymphocyte function-associated antigen 1; alpha polypeptide) |
| ITGAM | integrin, alpha M (complement component 3 receptor 3 subunit) |
| ITGAX | integrin, alpha X (complement component 3 receptor 4 subunit) |
| ITGB1 | integrin, beta 1 (fibronectin receptor, beta polypeptide, antigen CD29 includes MDF2, MSK12) |
| ITGB2 | integrin, beta 2 (complement component 3 receptor 3 and 4 subunit) |
| ITLN1 | intelectin 1 (galactofuranose binding) |
| ITLN2 | intelectin 2 |
| JAK1 | Janus kinase 1 |
| JAK2 | Janus kinase 2 |
| JAK3 | Janus kinase 3 |
| KCNJ2 | potassium inwardly-rectifying channel, subfamily J, member 2 |
| KIR_Activating_Subgroup_1 | killer cell immunoglobulin-like receptor |
| KIR_Activating_Subgroup_2 | killer cell immunoglobulin-like receptor |
| KIR_Inhibiting_Subgroup_1 | killer cell immunoglobulin-like receptor |
| KIR_Inhibiting_Subgroup_2 | killer cell immunoglobulin-like receptor |
| KIR3DL1 | killer cell immunoglobulin-like receptor, three domains, long cytoplasmic tail, 1 |
| KIR3DL2 | killer cell immunoglobulin-like receptor, three domains, long cytoplasmic tail, 2 |
| KIR3DL3 | killer cell immunoglobulin-like receptor, three domains, long cytoplasmic tail, 3 |
| KIT | v-kit Hardy-Zuckerman 4 feline sarcoma viral oncogene homolog |
| KLRAP1 | killer cell lectin-like receptor subfamily A pseudogene 1 |
| KLRB1 | killer cell lectin-like receptor subfamily B, member 1 |
| KLRC1 | killer cell lectin-like receptor subfamily C, member 1 |
| KLRC2 | killer cell lectin-like receptor subfamily C, member 2 |
| KLRC3 | killer cell lectin-like receptor subfamily C, member 3 |
| KLRC4 | killer cell lectin-like receptor subfamily C, member 4 |
| KLRD1 | killer cell lectin-like receptor subfamily D, member 1 |
| KLRF1 | killer cell lectin-like receptor subfamily F, member 1 |
| KLRF2 | killer cell lectin-like receptor subfamily F, member 2 |
| KLRG1 | killer cell lectin-like receptor subfamily G, member 1 |
| KLRG2 | killer cell lectin-like receptor subfamily G, member 2 |
| KLRK1 | killer cell lectin-like receptor subfamily K, member 1 |
| LAG3 | lymphocyte-activation gene 3 |
| LAIR1 | leukocyte-associated immunoglobulin-like receptor 1 |
| LAMP3 | lysosomal-associated membrane protein 3 |
| LCK | lymphocyte-specific protein tyrosine kinase |
| LCP2 | lymphocyte cytosolic protein 2 (SH2 domain containing leukocyte protein of 76kDa) |
| LEF1 | lymphoid enhancer-binding factor 1 |
| LGALS3 | lectin, galactoside-binding, soluble, 3 |
| LIF | leukemia inhibitory factor (cholinergic differentiation factor) |
| LILRA1 | leukocyte immunoglobulin-like receptor, subfamily A (with TM domain), member 1 |
| LILRA2 | leukocyte immunoglobulin-like receptor, subfamily A (with TM domain), member 2 |
| LILRA3 | leukocyte immunoglobulin-like receptor, subfamily A (without TM domain), member 3 |
| LILRA4 | leukocyte immunoglobulin-like receptor, subfamily A (with TM domain), member 4 |
| LILRA5 | leukocyte immunoglobulin-like receptor, subfamily A (with TM domain), member 5 |
| LILRA6 | leukocyte immunoglobulin-like receptor, subfamily A (with TM domain), member 6 |
| LILRB1 | leukocyte immunoglobulin-like receptor, subfamily B (with TM and ITIM domains), member 1 |
| LILRB2 | leukocyte immunoglobulin-like receptor, subfamily B (with TM and ITIM domains), member 2 |
| LILRB3 | leukocyte immunoglobulin-like receptor, subfamily B (with TM and ITIM domains), member 3 |
| LILRB4 | leukocyte immunoglobulin-like receptor, subfamily B (with TM and ITIM domains), member 4 |
| LILRB5 | leukocyte immunoglobulin-like receptor, subfamily B (with TM and ITIM domains), member 5 |
| LITAF | lipopolysaccharide-induced TNF factor |
| LTA | lymphotoxin alpha (TNF superfamily, member 1) |
| LTB4R | leukotriene B4 receptor |
| LTB4R2 | leukotriene B4 receptor 2 |
| LTBR | lymphotoxin beta receptor (TNFR superfamily, member 3) |
| LTF | lactotransferrin |
| LY96 | lymphocyte antigen 96 |
| MAF | v-maf musculoaponeurotic fibrosarcoma oncogene homolog (avian) |
| MALT1 | mucosa associated lymphoid tissue lymphoma translocation gene 1 |
| MAP4K1 | mitogen-activated protein kinase kinase kinase kinase 1 |
| MAP4K2 | mitogen-activated protein kinase kinase kinase kinase 2 |
| MAP4K4 | mitogen-activated protein kinase kinase kinase kinase 4 |
| MAPK1 | mitogen-activated protein kinase 1 |
| MAPK11 | mitogen-activated protein kinase 11 |
| MAPK14 | mitogen-activated protein kinase 14 |
| MAPKAPK2 | mitogen-activated protein kinase-activated protein kinase 2 |
| MARCO | macrophage receptor with collagenous structure |
| MASP1 | mannan-binding lectin serine peptidase 1 (C4/C2 activating component of Ra-reactive factor) |
| MASP2 | mannan-binding lectin serine peptidase 2 |
| MBL2 | mannose-binding lectin (protein C) 2, soluble |
| MBP | myelin basic protein |
| MCL1 | myeloid cell leukemia sequence 1 (BCL2-related) |
| MIF | macrophage migration inhibitory factor (glycosylation-inhibiting factor) |
| MME | membrane metallo-endopeptidase |
| MR1 | major histocompatibility complex, class I-related |
| MRC1 | mannose receptor, C type 1 |
| MS4A1 | membrane-spanning 4-domains, subfamily A, member 1 |
| MSR1 | macrophage scavenger receptor 1 |
| MUC1 | mucin 1, cell surface associated |
| MX1 | myxovirus (influenza virus) resistance 1, interferon-inducible protein p78 (mouse) |
| MYD88 | myeloid differentiation primary response gene (88) |
| NCAM1 | neural cell adhesion molecule 1 |
| NCF4 | neutrophil cytosolic factor 4, 40kDa |
| NCR1 | natural cytotoxicity triggering receptor 1 |
| NFATC1 | nuclear factor of activated T-cells, cytoplasmic, calcineurin-dependent 1 |
| NFATC2 | nuclear factor of activated T-cells, cytoplasmic, calcineurin-dependent 2 |
| NFATC3 | nuclear factor of activated T-cells, cytoplasmic, calcineurin-dependent 3 |
| NFIL3 | nuclear factor, interleukin 3 regulated |
| NFKB1 | nuclear factor of kappa light polypeptide gene enhancer in B-cells 1 |
| NFKB2 | nuclear factor of kappa light polypeptide gene enhancer in B-cells 2 (p49/p100) |
| NFKBIA | nuclear factor of kappa light polypeptide gene enhancer in B-cells inhibitor, alpha |
| NFKBIZ | nuclear factor of kappa light polypeptide gene enhancer in B-cells inhibitor, zeta |
| NLRP3 | NLR family, pyrin domain containing 3 |
| NOD1 | nucleotide-binding oligomerization domain containing 1 |
| NOD2 | nucleotide-binding oligomerization domain containing 2 |
| NOS2 | nitric oxide synthase 2, inducible |
| NOTCH1 | notch 1 |
| NOTCH2 | notch 2 |
| NT5E | 5'-nucleotidase, ecto (CD73) |
| PAX5 | paired box 5 |
| PDCD1 | programmed cell death 1 |
| PDCD1LG2 | programmed cell death 1 ligand 2 |
| PDCD2 | programmed cell death 2 |
| PDGFB | platelet-derived growth factor beta polypeptide |
| PDGFRB | platelet-derived growth factor receptor, beta polypeptide |
| PECAM1 | platelet/endothelial cell adhesion molecule |
| PIGR | polymeric immunoglobulin receptor |
| PLA2G2A | phospholipase A2, group IIA (platelets, synovial fluid) |
| PLA2G2E | phospholipase A2, group IIE |
| PLAU | plasminogen activator, urokinase |
| PLAUR | plasminogen activator, urokinase receptor |
| PML | promyelocytic leukemia |
| POU2F2 | POU class 2 homeobox 2 |
| PPARG | peroxisome proliferator-activated receptor gamma |
| PPBP | pro-platelet basic protein (chemokine (C-X-C motif) ligand 7) |
| PRDM1 | PR domain containing 1, with ZNF domain |
| PRF1 | perforin 1 (pore forming protein) |
| PRKCD | protein kinase C, delta |
| PSMB10 | proteasome (prosome, macropain) subunit, beta type, 10 |
| PSMB5 | proteasome (prosome, macropain) subunit, beta type, 5 |
| PSMB7 | proteasome (prosome, macropain) subunit, beta type, 7 |
| PSMB8 | proteasome (prosome, macropain) subunit, beta type, 8 (large multifunctional peptidase 7) |
| PSMB9 | proteasome (prosome, macropain) subunit, beta type, 9 (large multifunctional peptidase 2) |
| PSMC2 | proteasome (prosome, macropain) 26S subunit, ATPase, 2 |
| PSMD7 | proteasome (prosome, macropain) 26S subunit, non-ATPase, 7 |
| PTAFR | platelet-activating factor receptor |
| PTGER4 | prostaglandin E receptor 4 (subtype EP4) |
| PTGS2 | prostaglandin-endoperoxide synthase 2 (prostaglandin G/H synthase and cyclooxygenase) |
| PTK2 | PTK2 protein tyrosine kinase 2 |
| PTPN2 | protein tyrosine phosphatase, non-receptor type 2 |
| PTPN22 | protein tyrosine phosphatase, non-receptor type 22 (lymphoid) |
| PTPN6 | protein tyrosine phosphatase, non-receptor type 6 |
| PTPRC_all | protein tyrosine phosphatase, receptor type, C |
| CD45R0 | protein tyrosine phosphatase, receptor type, C |
| CD45RA | protein tyrosine phosphatase, receptor type, C |
| CD45RB | protein tyrosine phosphatase, receptor type, C |
| PYCARD | PYD and CARD domain containing |
| RAF1 | v-raf-1 murine leukemia viral oncogene homolog 1 |
| RAG1 | recombination activating gene 1 |
| RAG2 | recombination activating gene 2 |
| RARRES3 | retinoic acid receptor responder (tazarotene induced) 3 |
| RELA | v-rel reticuloendotheliosis viral oncogene homolog A (avian) |
| RELB | v-rel reticuloendotheliosis viral oncogene homolog B |
| RORC | RAR-related orphan receptor C |
| RUNX1 | runt-related transcription factor 1 |
| S100A8 | S100 calcium binding protein A8 |
| S100A9 | S100 calcium binding protein A9 |
| S1PR1 | sphingosine-1-phosphate receptor 1 |
| SELE | selectin E |
| SELL | selectin L |
| SELPLG | selectin P ligand |
| SERPING1 | serpin peptidase inhibitor, clade G (C1 inhibitor), member 1 |
| SH2D1A | SH2 domain containing 1A |
| SIGIRR | single immunoglobulin and toll-interleukin 1 receptor (TIR) domain |
| SKI | v-ski sarcoma viral oncogene homolog (avian) |
| SLAMF1 | signalling lymphocytic activation molecule family member 1 |
| SLAMF6 | SLAM family member 6 |
| SLAMF7 | SLAM family member 7 |
| SLC2A1 | solute carrier family 2 (facilitated glucose transporter), member 1 |
| SMAD3 | SMAD family member 3 |
| SMAD5 | SMAD family member 5 |
| SOCS1 | suppressor of cytokine signalling 1 |
| SOCS3 | suppressor of cytokine signalling 3 |
| SPP1 | secreted phosphoprotein 1 |
| SRC | v-src sarcoma (Schmidt-Ruppin A-2) viral oncogene homolog (avian) |
| STAT1 | signal transducer and activator of transcription 1, 91kDa |
| STAT2 | signal transducer and activator of transcription 2, 113kDa |
| STAT3 | signal transducer and activator of transcription 3 (acute-phase response factor) |
| STAT4 | signal transducer and activator of transcription 4 |
| STAT5A | signal transducer and activator of transcription 5A |
| STAT5B | signal transducer and activator of transcription 5B |
| STAT6 | signal transducer and activator of transcription 6, interleukin-4 induced |
| SYK | spleen tyrosine kinase |
| TAGAP | T-cell activation RhoGTPase activating protein |
| TAL1 | T-cell acute lymphocytic leukemia 1 |
| TAP1 | transporter 1, ATP-binding cassette, sub-family B (MDR/TAP) |
| TAP2 | transporter 2, ATP-binding cassette, sub-family B (MDR/TAP) |
| TAPBP | TAP binding protein (tapasin) |
| TBK1 | TANK-binding kinase 1 |
| TBX21 | T-box 21 |
| TCF4 | transcription factor 4 |
| TCF7 | transcription factor 7 (T-cell specific, HMG-box) |
| TFRC | transferrin receptor (p90, CD71) |
| TGFB1 | transforming growth factor, beta 1 |
| TGFBI | transforming growth factor, beta-induced, 68kDa |
| TGFBR1 | transforming growth factor, beta receptor 1 |
| TGFBR2 | transforming growth factor, beta receptor II (70/80kDa) |
| THY1 | Thy-1 cell surface antigen |
| TICAM1 | toll-like receptor adaptor molecule 1 |
| TIGIT | T cell immunoreceptor with Ig and ITIM domains |
| TIRAP | toll-interleukin 1 receptor (TIR) domain containing adaptor protein |
| TLR1 | toll-like receptor 1 |
| TLR2 | toll-like receptor 2 |
| TLR3 | toll-like receptor 3 |
| TLR4 | toll-like receptor 4 |
| TLR5 | toll-like receptor 5 |
| TLR7 | toll-like receptor 7 |
| TLR8 | toll-like receptor 8 |
| TLR9 | toll-like receptor 9 |
| TMEM173 | transmembrane protein 173 |
| TNF | tumor necrosis factor |
| TNFAIP3 | tumor necrosis factor, alpha-induced protein 3 |
| TNFAIP6 | tumor necrosis factor, alpha-induced protein 6 |
| TNFRSF10C | tumor necrosis factor receptor superfamily, member 10c, decoy without an intracellular domain |
| TNFRSF11A | tumor necrosis factor receptor superfamily, member 11a, NFKB activator |
| TNFRSF13B | tumor necrosis factor receptor superfamily, member 13B |
| TNFRSF13C | tumor necrosis factor receptor superfamily, member 13C |
| TNFRSF14 | tumor necrosis factor receptor superfamily, member 14 |
| TNFRSF17 | tumor necrosis factor receptor superfamily, member 17 |
| TNFRSF1B | tumor necrosis factor receptor superfamily, member 1B |
| TNFRSF4 | tumor necrosis factor receptor superfamily, member 4 |
| TNFRSF8 | tumor necrosis factor receptor superfamily, member 8 |
| TNFRSF9 | tumor necrosis factor receptor superfamily, member 9 |
| TNFSF10 | tumor necrosis factor (ligand) superfamily, member 10 |
| TNFSF11 | tumor necrosis factor (ligand) superfamily, member 11 |
| TNFSF12 | tumor necrosis factor (ligand) superfamily, member 12 |
| TNFSF13B | tumor necrosis factor (ligand) superfamily, member 13b |
| TNFSF15 | tumor necrosis factor (ligand) superfamily, member 15 |
| TNFSF4 | tumor necrosis factor (ligand) superfamily, member 4 |
| TNFSF8 | tumor necrosis factor (ligand) superfamily, member 8 |
| TOLLIP | toll interacting protein |
| TP53 | tumor protein p53 |
| TRAF1 | TNF receptor-associated factor 1 |
| TRAF2 | TNF receptor-associated factor 2 |
| TRAF3 | TNF receptor-associated factor 3 |
| TRAF4 | TNF receptor-associated factor 4 |
| TRAF5 | TNF receptor-associated factor 5 |
| TRAF6 | TNF receptor-associated factor 6 |
| TYK2 | tyrosine kinase 2 |
| UBE2L3 | ubiquitin-conjugating enzyme E2L 3 |
| VCAM1 | vascular cell adhesion molecule 1 |
| VTN | vitronectin |
| XBP1 | X-box binding protein 1 |
| XCL1 | chemokine (C motif) ligand 1 |
| XCR1 | chemokine (C motif) receptor 1 |
| ZAP70 | zeta-chain (TCR) associated protein kinase 70kDa |
| ZBTB16 | zinc finger and BTB domain containing 16 |
| ZEB1 | zinc finger E-box binding homeobox 1 |

**Supplementary Note 2. Primary annotations of genes profiled from the Nanostring Human Immunology panel.**

| **Primary Annotation** | **Number of genes** |
| --- | --- |
| Adaptive Immune System | 141 |
| Apoptosis | 54 |
| Autophagy | 11 |
| B cell Receptor Signalling | 35 |
| Cell Adhesion | 60 |
| Chemokine Signalling | 63 |
| Complement System | 39 |
| Cytokine Signalling | 259 |
| Haemostasis | 73 |
| Host-pathogen Interaction | 252 |
| Immunometabolism | 32 |
| Inflammasomes | 8 |
| Innate Immune System | 201 |
| Lymphocyte Activation | 245 |
| Lymphocyte Trafficking | 21 |
| MHC Class I Antigen Presentation | 39 |
| MHC Class II Antigen Presentation | 14 |
| NF-kB Signalling | 62 |
| NLR signalling | 64 |
| Oxidative Stress | 36 |
| Phagocytosis and Degradation | 48 |
| T Cell Receptor Signalling | 61 |
| TGF-b Signalling | 9 |
| Th1 Differentiation | 14 |
| Th17 Differentiation | 31 |
| Th2 Differentiation | 17 |
| TNF Family Signalling | 49 |
| TLR Signalling | 73 |
| Transcriptional Regulation | 53 |
| Treg Differentiation | 10 |
| Type I Interferon Signalling | 28 |
| Type II Interferon Signalling | 36 |

**Supplementary Note 3. Metabolites investigated using ^1^H-nuclear magnetic resonance spectroscopy.**

| **TPTG** |
| --- |
| **TPCH** |
| **LDCH** |
| **HDCH** |
| **TPA1** |
| **TPA2** |
| **TPAB** |
| **LDHD** |
| **ABA1** |
| **TBPN** |
| **VLPN** |
| **IDPN** |
| **LDPN** |
| **L1PN** |
| **L2PN** |
| **L3PN** |
| **L4PN** |
| **L5PN** |
| **L6PN** |
| **VLTG** |
| **IDTG** |
| **LDTG** |
| **HDTG** |
| **VLCH** |
| **IDCH** |
| **VLFC** |
| **IDFC** |
| **LDFC** |
| **HDFC** |
| **VLPL** |
| **IDPL** |
| **LDPL** |
| **HDPL** |
| **HDA1** |
| **HDA2** |
| **VLAB** |
| **IDAB** |
| **LDAB** |
| **V1TG** |
| **V2TG** |
| **V3TG** |
| **V4TG** |
| **V5TG** |
| **V1CH** |
| **V2CH** |
| **V3CH** |
| **V4CH** |
| **V5CH** |
| **V1FC** |
| **V2FC** |
| **V3FC** |
| **V4FC** |
| **V5FC** |
| **V1PL** |
| **V2PL** |
| **V3PL** |
| **V4PL** |
| **V5PL** |
| **L1TG** |
| **L2TG** |
| **L3TG** |
| **L4TG** |
| **L5TG** |
| **L6TG** |
| **L1CH** |
| **L2CH** |
| **L3CH** |
| **L4CH** |
| **L5CH** |
| **L6CH** |
| **L1FC** |
| **L2FC** |
| **L3FC** |
| **L4FC** |
| **L5FC** |
| **L6FC** |
| **L1PL** |
| **L2PL** |
| **L3PL** |
| **L4PL** |
| **L5PL** |
| **L6PL** |
| **L1AB** |
| **L2AB** |
| **L3AB** |
| **L4AB** |
| **L5AB** |
| **L6AB** |
| **H1TG** |
| **H2TG** |
| **H3TG** |
| **H4TG** |
| **H1CH** |
| **H2CH** |
| **H3CH** |
| **H4CH** |
| **H1FC** |
| **H2FC** |
| **H3FC** |
| **H4FC** |
| **H1PL** |
| **H2PL** |
| **H3PL** |
| **H4PL** |
| **H1A1** |
| **H2A1** |
| **H3A1** |
| **H4A1** |
| **H1A2** |
| **H2A2** |
| **H3A2** |
| **H4A2** |
| **Ethanol** |
| **Trimethylamine-N-oxide** |
| **2-Aminobutyric acid** |
| **Alanine** |
| **Asparagine** |
| **Creatine** |
| **Creatinine** |
| **Glutamic acid** |
| **Glutamine** |
| **Glycine** |
| **Histidine** |
| **Isoleucine** |
| **Leucine** |
| **Lysine** |
| **Methionine** |
| **N,N-Dimethylglycine** |
| **Ornithine** |
| **Phenylalanine** |
| **Proline** |
| **Sarcosine** |
| **Threonine** |
| **Tyrosine** |
| **Valine** |
| **2-Hydroxybutyric acid** |
| **Acetic acid** |
| **Citric acid** |
| **Formic acid** |
| **Lactic acid** |
| **Succinic acid** |
| **Choline** |
| **2-Oxoglutaric acid** |
| **3-Hydroxybutyric acid** |
| **Acetoacetic acid** |
| **Acetone** |
| **Pyruvic acid** |
| **D-Galactose** |
| **Glucose** |
| **Glycerol** |
| **Dimethylsulfone** |
| **Ca-EDTA** |
| **K-EDTA** |

**Supplementary Figure 1. Heatmap of 579 gene transcripts from the Nanostring Human Immunology panel on the baseline biopsies of patients achieving best response - responders (n=3) and non-responders (n=3).**


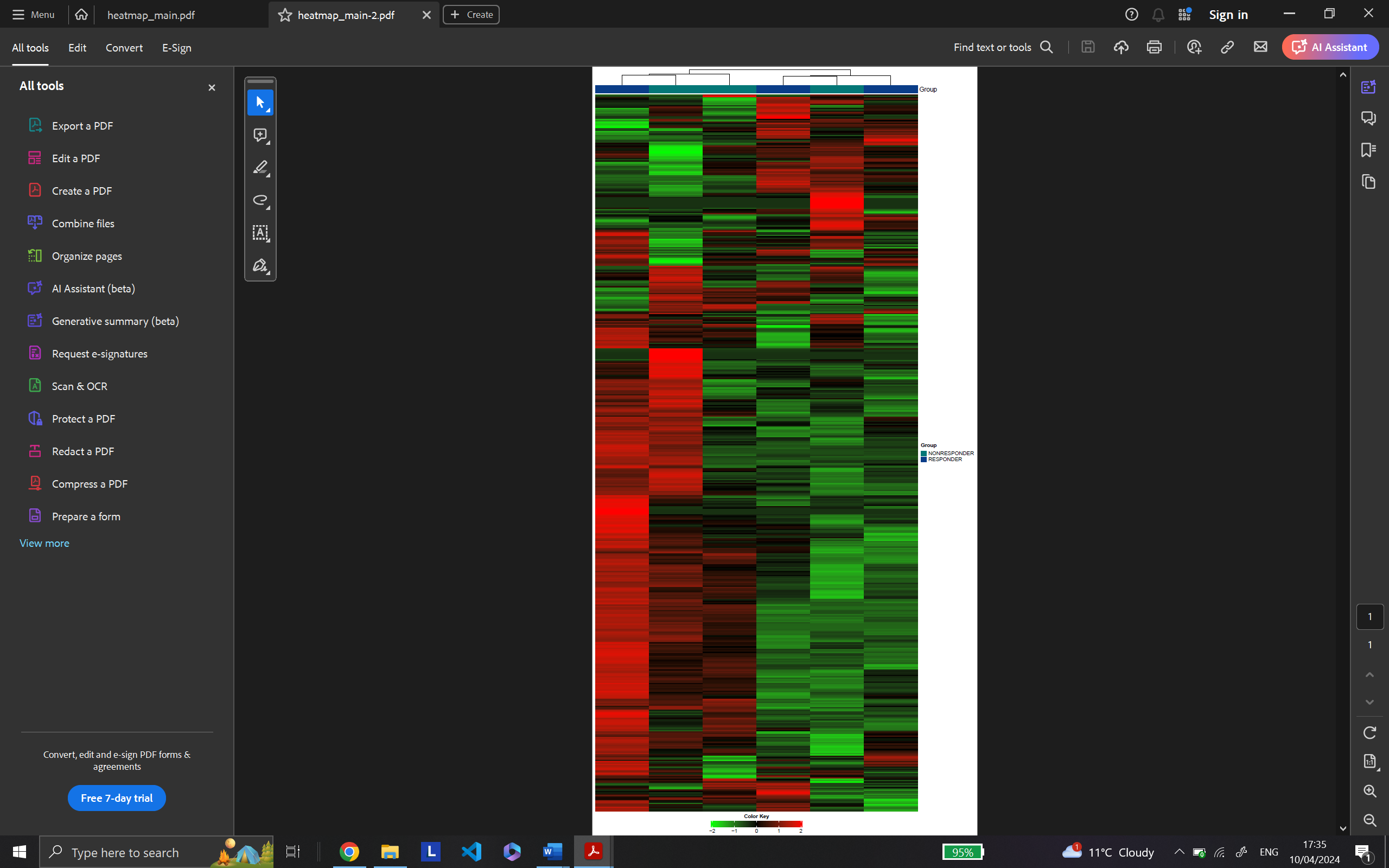


**Supplementary Figure 2. Gene set enrichment analysis for the differential expression of 32 gene signatures between responders and non-responders.**


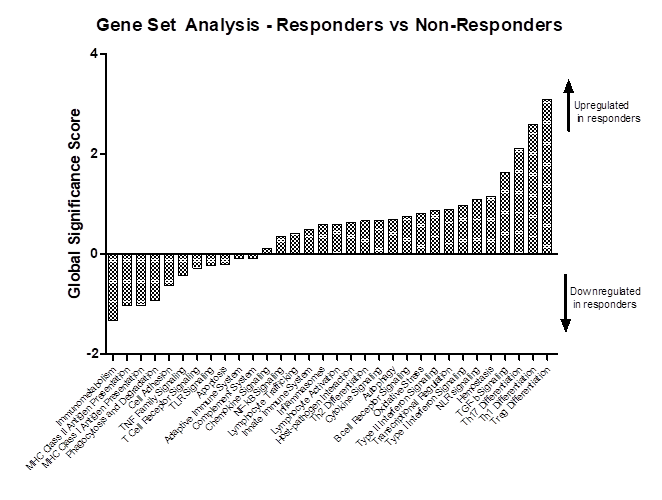


**Supplementary Figure 3. Distribution of normalized concentrations across all samples**.


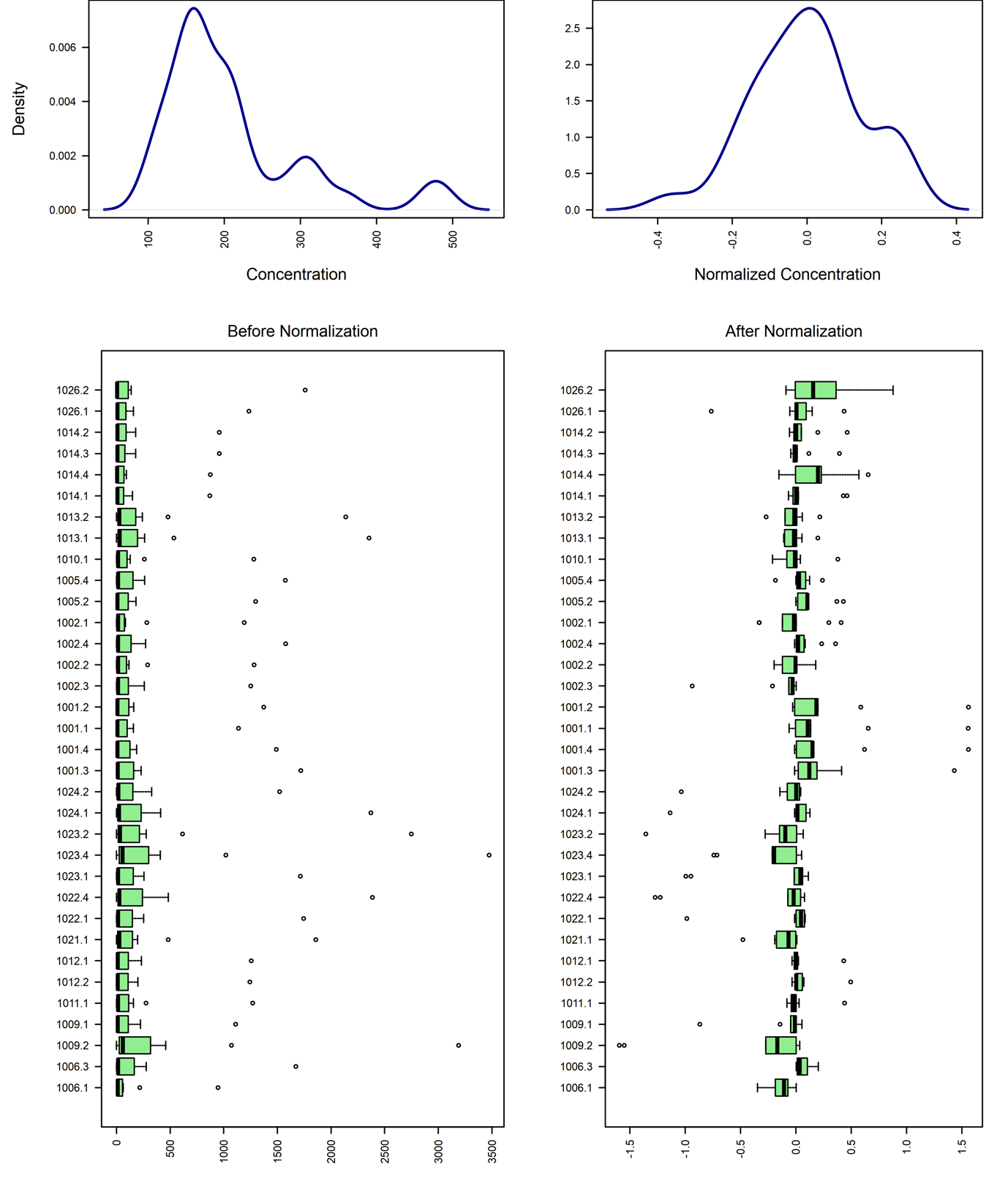


**Supplementary Figure 4. Metabolite concentrations after normalization**. Normalized concentrations of individual metabolites, with green boxes indicating the interquartile range, the black line within the boxes representing the median, and the whiskers showing 1.5 times the interquartile range.


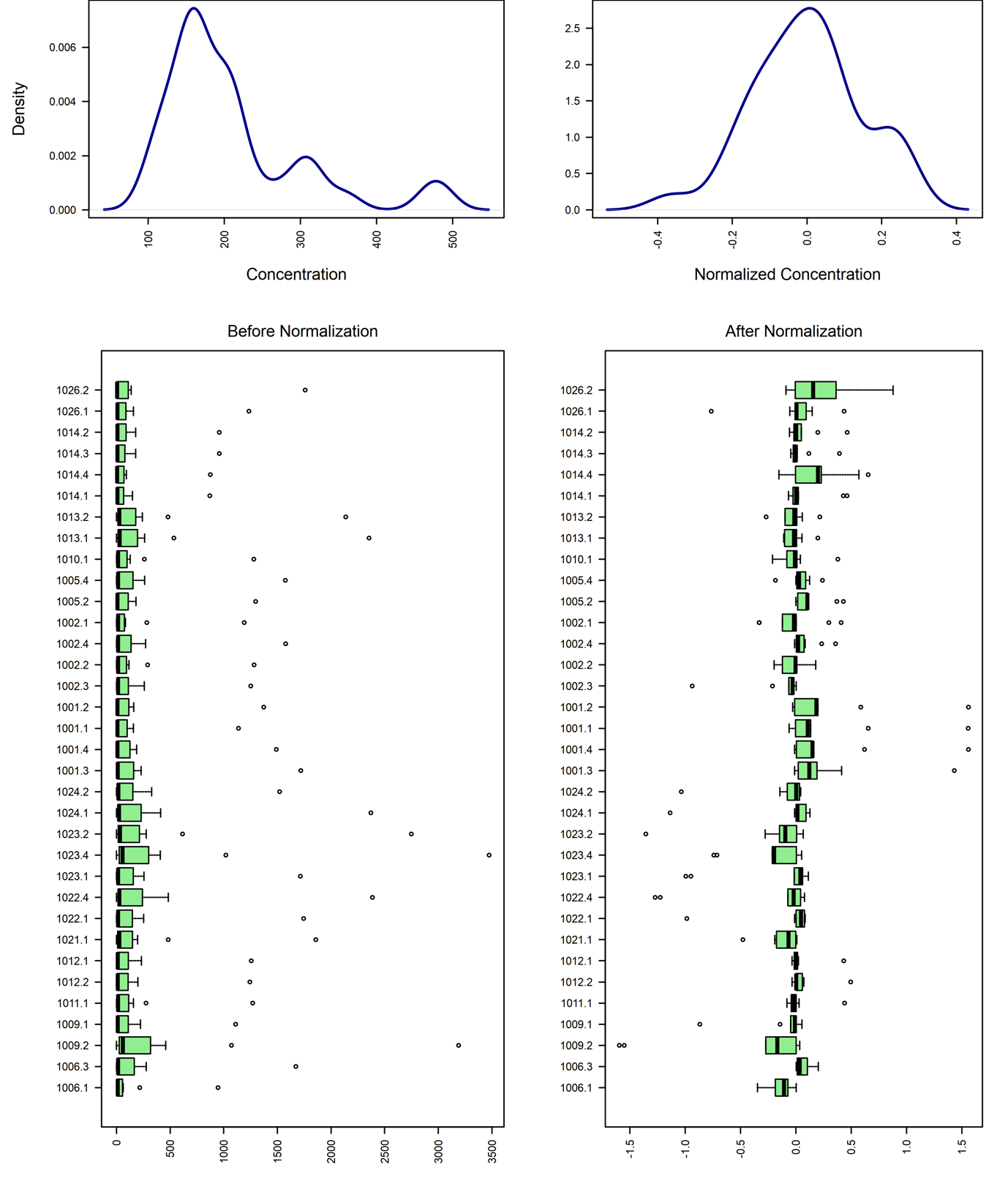


**Supplementary Figure 5. Box plots of proline in responders across different treatment cycles illustrating an initial reduction in metabolites in responders**. The green line represents the median value, and the black lines are the interquartile ranges.


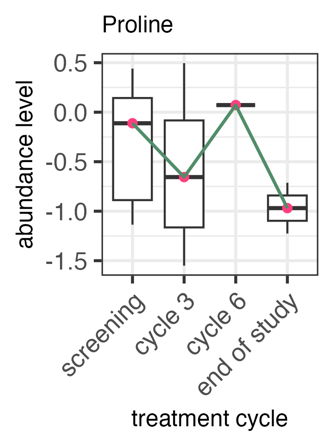


**Supplementary Figure 6. Box plots of proline in non-responders illustrating no change in those not experiencing a response**. The green line represents the median value, and the black lines are the interquartile ranges.


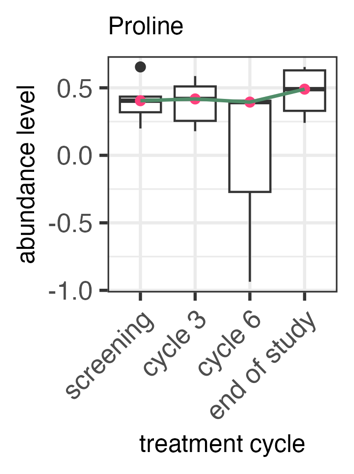


**Supplementary Figure 7. Box plots of acetic acid in responders across different treatment cycles illustrating an initial reduction in metabolites in responders**. The green line represents the median value, and the black lines are the interquartile ranges.


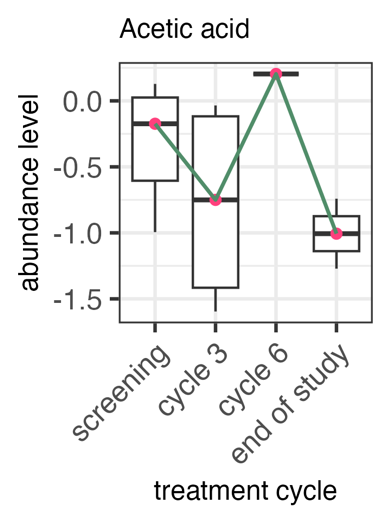


**Supplementary Figure 8. Box plots of acetic acid in non-responders across different treatment cycles illustrating no change in those not experiencing a response**. The green line represents the median value, and the black lines are the interquartile ranges.


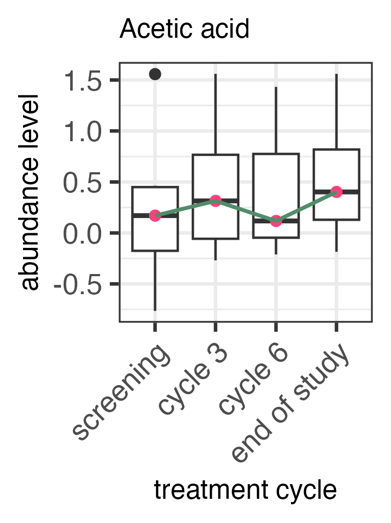

Supplement: Supplementary file 1 — Supplementary [file 41698_2026_1273_MOESM1_ESM.docx]
